# Stromal‐Derived IL‐8 Promotes M2 Macrophage Polarization via the RhoA/MRTF‐A/SRF Transcriptional Axis to Impair CD8^+^ T Cell Cytotoxicity in Lung Cancer

**DOI:** 10.1002/advs.202508001

**Published:** 2026-07-16

**Authors:** Xuyu Gu, Qiyu Fang, Jia Yu, Ting Yu, Huashan Shi, Kaiqi Jin, Lei Jiang, Wentian Zhang

**Affiliations:** ^1^ Department of Oncology School of Medicine Shanghai Pulmonary Hospital Tongji University Medical School Cancer Institute Tongji University Shanghai China; ^2^ Department of Biotherapy Cancer Center West China Hospital Sichuan University Chengdu China; ^3^ Department of Pathology and Laboratory of Pathology State Key Laboratory of Biotherapy West China School of Medicine West China Hospital Sichuan University Chengdu China; ^4^ Department of Thoracic Surgery Shanghai Pulmonary Hospital School of Medicine Tongji University Shanghai China

**Keywords:** cancer‐associated fibroblasts, lung cancer, tumor microenvironment, tumor progression, tumor‐associated macrophages

## Abstract

**Background:**

Building upon our previous finding that tumor‐derived lactate activates DESMIN^+^ cancer‐associated fibroblasts (CAFs) to secrete interleukin‐8 (IL‐8), this study investigates the downstream IL‐8/C‐X‐C motif chemokine receptor 2 (CXCR2) axis in CAF‐mediated macrophage polarization and lung cancer (LC) progression.

**Methods:**

Orthotopic and subcutaneous tumor models were established in immunocompetent and immunodeficient mice using LC, CAFs, or murine tumor organoids (MTO). Global and myeloid‐specific *Cxcr2* knockout models, in vitro co‐cultures (macrophages, CD8^+^ T cells, and tumor cells), and patient‐derived organoid (PDO) systems were employed.

**Results:**

DESMIN^+^ CAFs were the predominant source of IL‐8 and promoted tumorigenesis and M2‐like macrophage accumulation via the IL‐8/CXCR2 axis. Genetic deletion of *Cxcr2*, myeloid‐specific knockout, or pharmacological blockade reduced M2‐like macrophage polarization through the PI3K/AKT/RhoA/MRTF‐A/SRF pathway, restored CD8^+^ T cell effector function, and suppressed tumor progression. CXCR2 blockade also synergized with chemotherapy and anti‐PD‐L1 immunotherapy in murine models. In human PDO co‐cultures with matched DESMIN^+^ CAFs and autologous CD8^+^ T cells, CXCR2 inhibition attenuated M2‐like polarization and enhanced T cell‐mediated killing of tumor organoids.

**Conclusion:**

DESMIN^+^ CAFs drive an immunosuppressive tumor microenvironment in LC through IL‐8/CXCR2 signaling and downstream MRTF‐A activation. Targeting this axis represents a promising strategy to overcome treatment resistance in LC.

## Graphical abstract

In the recondite pulmonary neoplastic milieu, DESMIN^+^ cancer‐associated fibroblasts function as the paramount stromal source of IL‐8, precipitating a deleterious immunological metamorphosis by engaging CXCR2 receptors on the myeloid lineage. This paracrine stimulus triggers a mechanotransduction cascade characterized by RhoA‐dependent actin polymerization, which facilitates the quintessential liberation and subsequent nuclear translocation of the transcriptional co‐activator MRTF‐A from its cytoplasmic sequestration. Once localized within the nucleus, the MRTF‐A/SRF complex is situated in the promoters of Arg1 and Mrc1, thereby orchestrating the transcriptional reprogramming of macrophages into an immunosuppressive M2‐like phenotype. These reprogrammed myeloid cells engender a paradigmatic state of T‐cell exclusion and functional abrogation, shielding tumor clusters from cytotoxic surveillance through the induction of terminal lymphocyte exhaustion. Ultimately, pharmacological intervention with the CXCR2 antagonist SB225002 targets these polarized macrophages to abrogate the immunosuppressive axis, recalibrating the myeloid phenotype to reinstate robust anti‐tumor immunity and catalyze the targeted destruction of patient‐derived tumor organoids.

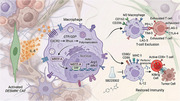

## 1 Introduction

In 2025, lung cancer (LC) stood as the most commonly diagnosed malignancy, accounting for nearly 2.1 million new cases, or approximately 11.09% of all cancers in the United States, translating to one in every eight cancer diagnoses [1]. LC is primarily classified into two main histological types: small cell lung cancer and non‐small cell lung cancer (NSCLC). The NSCLC subtype can be further subdivided into adenocarcinoma, squamous cell carcinoma, and large cell carcinoma, among others [2]. From a clinical perspective, only about 20%–25% of patients with NSCLC are diagnosed at an early stage (I or II) where surgical resection is possible. In contrast, a significant number of patients present with more advanced stages, for which treatment options typically include standard chemotherapy, radiation, targeted therapies, and immunotherapy [3, 4]. Unfortunately, despite advancements in therapeutic strategies, the prognosis remains poor for lung cancer patients. For those diagnosed with distant metastases, the overall five‐year survival rate plummets to a mere 5.5% [5].

Recent studies have highlighted the tumor microenvironment (TME) as a critical factor in tumor immune evasion, metastasis, local resistance, and response to targeted therapies [6, 7, 8]. The TME is a complex ecosystem comprised of tumor cells, various immune cells (such as macrophages, dendritic cells, NK cells, T cells, and B cells), cancer‐associated stromal cells (particularly cancer‐associated fibroblasts (CAFs), endothelial cells, adipocytes, along with an intricate extracellular matrix and numerous signaling molecules [9, 10]. CAFs are primary elements of the stroma surrounding tumors [11]. They contribute to fibrosis within the TME, leading to enhanced deposition and remodeling of the extracellular matrix, which can significantly obstruct anti‐tumor immune mechanisms [12]. The influence of CAFs on tumor development is likely multifaceted [13]; they may promote tumor growth by facilitating T cell exclusion through modified matrix production [14], enhancing cell survival [15, 16], and influencing inflammatory responses [17]. We recently found that lactate present in the TME of LC boosts CAF activation by increasing DESMIN expression [18]. Additionally, our preliminary investigations suggest that these activated CAFs may recruit tumor‐associated macrophages (TAMs) and alter their characteristics potentially via an interleukin (IL)‐8/IL8 receptor (IL‐8R) pathway [18]. Macrophages are critical immune cells playing core roles in both maintaining homeostasis and regulating disease processes, performing a wide range of tasks including engulfing pathogens, presenting antigens, and releasing signaling molecules [19, 20]. These cells are highly adaptable, and their numbers can quickly increase in response to injury, inflammation, or cancer, as monocytes migrate into affected tissues during such pathological conditions [21, 22, 23]. In the context of TME, TAMs are the most abundant tumor‐infiltrating immune cells, which are roughly divided into two major functional subtypes: classical activated M1 macrophages, which exhibit anti‐tumor activities, and alternatively activated M2 macrophages, which are associated with promoting tumor progression [24] M1 macrophages are activated by interferon (IFN)‐γ (generated by T‐helper 1 cells) and bacterial lipopolysaccharides, which are typically associated with pro‐inflammatory responses and anti‐tumor activity, producing inflammatory cytokines like IL‐1β, IL‐6, and tumor necrosis factor (TNF)‐α. On the other hand, M2 macrophages are activated by IL‐4 and IL‐13 (released by T‐helper 2 cells) and play a significant role in tumor development, growth, spread, and immune system avoidance. They primarily secrete anti‐inflammatory molecules like IL‐10 and transforming growth factor (TGF)‐β [25, 26, 27]. In the TME, M2‐like TAMs primarily support tumor advancement through mechanisms such as angiogenesis, immune suppression, and facilitated metastasis [24, 28, 29]. Although these M2‐like TAMs secrete immune checkpoint modulators that suppress T cell function, they also hold the potential for reprogramming into the M1 phenotype, which possesses anti‐tumor properties [30]. Therefore, efforts aimed at reprogramming TAMs toward an M1 phenotype present a promising avenue to counteract tumor immunosuppression. IL‐8 is a pro‐inflammatory CXC chemokine that influences an array of intracellular signaling pathways through two cell‐surface G protein‐coupled receptors [31]. Elevated levels of IL‐8 and its receptors have been observed in cancer cells, endothelial cells, and infiltrating immune cells. Its involvement in angiogenesis, tumor growth, metastasis, and tumor resistance to drugs has been well‐documented [31]. The IL‐8 signaling has been shown to promote the recruitment of TAMs and enhance their pro‐tumor properties [32, 33]. Grounded on the insights of previous findings of our and others, this study seeks to enhance the understanding of the role of DESMIN^+^ CAFs in regulating the accumulation and polarization of TAMs. Specifically, the involvement of the IL‐8 axis in the associated events will be explored.

## 2 Materials and Methods

### 2.1 Tissue Microarray (TMA) Analysis

For the TMA analysis, we utilized a lung cancer tissue microarray (LC TMA) purchased from Bioaitech (Shanghai, China), containing 118 human LC samples, including primary tumor tissues and adjacent normal tissues. The TMA slides were deparaffinized, rehydrated, and subjected to antigen retrieval using citrate buffer (pH 6.0) in a microwave. The sections were incubated with an anti‐DESMIN antibody (1:200 dilution, Abcam, ab32362) overnight at 4°C. Detection was performed using the EnVision^+^ System‐HRP (Dako), followed by DAB chromogen. The stained sections were scanned using a digital slide scanner (Leica Aperio AT2), and the staining intensity of DESMIN was scored by two independent pathologists. The scoring was based on both the staining intensity (graded 0–3) and the percentage of positive cells (0%–100%). The correlation between DESMIN staining intensity and clinicopathological features (tumor stage, lymph node metastasis, recurrence, and microsatellite stability) was analyzed.

### 2.2 Human Patient‐Derived Organoid (PDO) and Primary Cell Preparation

Human PDOs were established from surgically resected lung cancer tissues. Briefly, tumor fragments were enzymatically dissociated into cell clusters and embedded in 3D Matrigel (Corning) using human organoid growth media containing specific niche factors (EGF, FGF‐10, Noggin).

To obtain human primary macrophages, peripheral blood mononuclear cells (PBMCs) were isolated from patient blood using Ficoll density gradient centrifugation. CD14^+^ monocytes were enriched via magnetic‐activated cell sorting (MACS, Miltenyi Biotec) and differentiated into macrophages in the presence of 50 ng/mL human M‐CSF for 7 days. Human Desmin^+^ CAFs were isolated from matched peritumoral stroma using primary fibroblast culture techniques and validated by immunofluorescence.

### 2.3 Mice

*Cxcr2* deficient (*Cxcr2* ^−/−^, strain #002724) mice were procured from Jackson Laboratories, while C57BL/6 (B6) mice were acquired from Beijing SLC, Inc. Both mouse strains were bred within a B6 genetic background and were bred and housed under germ‐free conditions. These mice were maintained within a controlled environment characterized by a 14 h light and 10 h dark cycle, with temperature and humidity levels set at 24°C and 50%, respectively. All procedures involving the handling of animals and experimental protocols adhered to the institutional guidelines and received approval from the animal research committee of Tongji University, Shanghai, China. These procedures were conducted in compliance with the regulations stipulated by the National Institutes of Health (NIH). For the purpose of the experiments, male mice aged between 8 and 10 weeks were employed.

### 2.4 Cell Culture

Mouse lung cancer cell lines 3LL and KLN205 were obtained from the American Type Culture Collection (ATCC). RAW264.7 murine macrophages were also purchased from ATCC. eGFP‐DESMIN^+^ CAFs were generated from murine lung tumor tissues as described below. All cell lines were cultured in a humidified incubator at 37°C with 5% CO_2_. 3LL and KLN205 cells were cultured in Dulbecco's Modified Eagle Medium (DMEM; Gibco) supplemented with 10% fetal bovine serum (FBS; HyClone), 1% penicillin–streptomycin (Gibco), and 1% glutamine (Sigma‐Aldrich). Media were replaced every 2–3 d, and cells were passaged using 0.25% trypsin‐EDTA (Gibco) when they reached 70–80% confluence. For experimental use, cells were harvested at 70%–80% confluence and counted using a hemocytometer (Neubauer Improved, Germany). Viability was confirmed using Trypan Blue exclusion assay (Thermo Fisher), ensuring > 95% viable cells for injection. RAW264.7 macrophages were cultured in DMEM supplemented with 10% FBS, 1% penicillin‐streptomycin, and 1% glutamine. Cells were seeded at 1 × 10^5^ cells/well in 6‐well plates and cultured for 24 h before being co‐cultured with CAFs or treated with recombinant IL‐8 (rIL‐8, PeproTech) or SB225002 (Sigma‐Aldrich) as described in the co‐culture experiments. CAFs were isolated from subcutaneous murine lung tumor tissues. Tumor tissues were minced and digested with a solution containing 2 mg/mL collagenase II (Worthington Biochemical Corporation) and 0.5 mg/mL hyaluronidase (Sigma‐Aldrich) for 1 h at 37°C with constant shaking. The digested tissue was filtered through a 70 µm strainer and centrifuged at 300 *× g* for 5 min. Cells were resuspended in DMEM with 10% FBS and cultured for 5–7 d. CAF identity was confirmed by immunofluorescence staining for CAF markers Vimentin (1:200, Abcam) and Fibronectin (1:200, Abcam).

For experiments involving genetic modification, cells were transfected using Lipofectamine 3000 (Thermo Fisher) according to the manufacturer's protocol. For knockdown experiments, short hairpin RNA (shRNA) targeting *Cxcr2* was cloned into a pLKO.1 vector and transfected into 3LL or RAW264.7 cells. Cells were selected with 2 µg/mL puromycin (Sigma‐Aldrich) for stable cell line generation. The efficiency of knockdown was confirmed by RT‐qPCR and western blot (WB) analysis.

For primary murine CAF isolation, lung tumor tissues were minced and digested with 2 mg/mL collagenase II and 0.5 mg/mL hyaluronidase (Sigma) at 37°C for 1 h. Single‐cell suspensions were filtered through a 70 µm strainer. Desmin^+^ and Desmin^−^ CAFs were identified and sorted via fluorescence‐activated cell sorting based on their expression profiles of Vimentin, Fibronectin, alpha‐SMA, and FAP.

### 2.5 Animal Experiments

Murine lung cancer 3LL cells and CAFs were cultured in DMEM supplemented with 10% fetal bovine serum (FBS) and 1% penicillin/streptomycin. CAFs were isolated from tumor tissues and confirmed by immunostaining for CAF markers (VIM and FN1). To generate isograft models, 3LL cells were mixed with CAFs at a 3:1 ratio and subcutaneously injected into B6 mice. A control group received only 3LL cells. Tumor growth was monitored, and samples were collected for analysis of IL‐8 and CXCR2 expression in primary and metastatic tumors using qPCR and immunohistochemistry (IHC).

To investigate the role of CXCR2, eGFP‐DESMIN‐expressing CAFs were isolated and confirmed by immunostaining for DESMIN. 3LL cells were mixed with these eGFP‐DESMIN^+^ CAFs at a 3:1 ratio and injected via tail vein into wild‐type (WT) or *Cxcr2* ^−/−^ (#S‐KO‐01535, Cyagen) B6 mice. Tumor progression and metastasis were assessed in lung and liver tissues, with tumor nodules counted and immune cell infiltration analyzed. Additionally, murine tumor organoids (MTOs) were prepared and orthotopically implanted into the lung parenchyma of B6 mice. Post‐implantation, mice were treated with SB225002 or Navarixin (IL‐8R inhibitors), and tumor growth was monitored. Tumors, liver metastases, and lymph nodes were harvested for histological and immunofluorescence analysis, focusing on TAM (CD206^+^Ly6C^+^ cells) infiltration. Bone marrow samples were collected to evaluate the presence of CXCR2^+^CD206^+^Ly6C^+^ cells using flow cytometry.

In another subcutaneous mouse model, 3LL cells were injected subcutaneously into WT, *Cxcr2* ^fl/fl^ (#S‐CKO‐01765, Cyagen), and myeloid‐specific *Cxcr2* knock‐out (*Cxcr2* ^Cd11b‐KO^) mice. After 4 weeks, tumors were harvested and re‐implanted into the right lung lobes. Tumor progression and immune cell infiltration were monitored. In a separate experiment, MTOs were implanted into WT and *Cxcr2* ^Cd11b‐KO^ mice to assess CXCR2's role in myeloid cells. Tumor growth, TAM infiltration, immune checkpoint expression (PD‐1, PD‐L1, CTLA4, Galectin‐9), and CD8^+^ T cell cytotoxicity were evaluated using IHC and flow cytometry. Bone marrow‐derived macrophages (BMDMs) from *Cxcr2* ^Cd11b‐KO^ or *Cxcr2* ^fl/fl^ mice were induced into a TAM‐like phenotype using IL‐4 and macrophage colony‐stimulating factor (M‐CSF). These TAMs were co‐injected with 3LL cells into B6 mice to monitor tumor growth and TAM impact. Tumors were harvested for IHC to assess proliferative markers (Ki67^+^ and PCNA^+^ cells). TAMs isolated from 3LL tumors in *Cxcr2* ^Cd11b‐KO^ or *Cxcr2* ^fl/fl^ mice were used to co‐culture with naive CD8^+^ T cells in Transwell systems, assessing their effect on CD8^+^ T cell activation and cytotoxicity.

In orthotopic LC models, KLN205 lung cancer cells were injected intra‐pleurally into B6 mice, and conditioned medium (CM) of *Cxcr2* ^f1/f1^ or *Cxcr2* ^Cd11b‐KO^ mice‐derived TAMs was injected into the pleural cavity. Tumor progression was evaluated by measuring metastatic foci and tumor diameters. The role of CXCR2 in TAM‐mediated metastasis was further evaluated using a splenic‐liver metastasis model, where luciferase‐expressing KLN205 cells were injected into the spleen, and metastasis was tracked using bioluminescence imaging.

To determine whether the tumor‐promoting effects of *Cxcr2*‐expressing TAMs depend on CD8^+^ T cells, systemic CD8^+^ T cell depletion was performed in the subcutaneous KLN205 tumor model using an anti‐CD8 antibody. Mice were treated with either control IgG or anti‐CD8 antibodies, and the effect of TAM‐derived CM from *Cxcr2* ^f1/f1^ or *Cxcr2* ^Cd11b‐KO^ mice on tumor growth was assessed. Tumor growth rates were compared between the groups with or without CD8^+^ T cell depletion. The tumor inhibition rate was calculated in both IgG‐treated and CD8‐depleted groups to evaluate the extent to which *Cxcr2*‐expressing TAMs promote tumor growth through CD8^+^ T cell suppression.

Additionally, to assess the impact of CXCR2 antagonism, SB225002 was administered to 3LL‐bearing B6 mice via intraperitoneal injection. Tumor growth, immune cell infiltration, and response to combination therapies with carboplatin or anti‐PD‐L1 monoclonal antibodies were evaluated. The combined treatments showed enhanced inhibition of tumor growth compared to monotherapies, indicating the potential of SB225002 as an adjuvant in lung cancer therapy.

### 2.6 Bioluminescence Imaging

To assess tumor burden in vivo, bioluminescence imaging was conducted one day prior to the euthanasia of experimental animals. In a succinct description of the procedure, mice received an intraperitoneal injection of d‐luciferin (Caliper Life Sciences) at a dosage of 150 mg/kg. Subsequently, images were acquired within a time frame of 10 to 20 min post‐injection utilizing the IVIS Lumina‐II imaging system by PerkinElmer Life Sciences, employing a capture time of 1.0 min and medium binning settings. The Living Image version 4.0 software was utilized for the post‐processing of the acquired images. Signal intensity was quantified as the summation of all detected photon counts within the region of interest, following the subtraction of background luminescence.

### 2.7 Histological Analyses

For histological analysis, tumor, lung, and liver tissues were excised and immediately fixed in 10% neutral buffered formalin (NBF; Sigma‐Aldrich) for 24–48 h at room temperature. Tissues were then dehydrated using an automated tissue processor (Leica TP1020), embedded in paraffin blocks using a paraffin embedding station (Leica EG1150), and sectioned into 5 µm thick slices using a rotary microtome (Leica RM2235). The sections were mounted onto glass slides (Superfrost Plus, Thermo Fisher Scientific) and dried at 60°C for 1 h. Hematoxylin and eosin (H&E) staining was performed to evaluate tissue morphology: slides were deparaffinized in xylene for 10 min, rehydrated through a graded ethanol series (100%, 95%, 70%), and stained with Harris hematoxylin (Sigma‐Aldrich) for 5 min, followed by eosin (Sigma‐Aldrich) for 2 min. Sections were then dehydrated through graded alcohols, cleared in xylene, and coverslipped using DPX mounting medium (Sigma‐Aldrich). Images were captured using a light microscope (Nikon Eclipse Ci‐L) at 20× magnification, and digital images were processed with NIS‐Elements software (Nikon). For the evaluation of stromal fibrosis, Masson's trichrome staining was performed using a commercially available kit (Sigma‐Aldrich) following the manufacturer's protocol. Briefly, sections were rehydrated, stained with Weigert's iron hematoxylin for 10 min, followed by Biebrich scarlet‐acid fuchsin for 10 min, differentiated in phosphomolybdic/phosphotungstic acid, and stained with aniline blue for 5 min. Collagen fibers appeared blue, while nuclei stained black and cytoplasm red. Sections were imaged using a Nikon Ti2 microscope, and collagen deposition was quantified using ImageJ software by calculating the percentage area stained.

### 2.8 IHC

Paraffin‐embedded tissues were sectioned at 5 µm, deparaffinized in xylene, and rehydrated in graded ethanol solutions. Antigen retrieval was performed by immersing the slides in sodium citrate buffer (pH 6.0) or Tris‐EDTA buffer (pH 9.0), followed by heating in a microwave oven for 20 min or using a pressure cooker (BioCare Medical) set to 95°C for 15 min. After cooling, slides were rinsed in distilled water and incubated with 3% hydrogen peroxide for 10 min at room temperature to block endogenous peroxidase activity. Non‐specific binding was blocked by incubating sections with 5% bovine serum albumin (BSA; Sigma‐Aldrich) or 10% normal goat serum (Vector Laboratories) for 1 h at room temperature. Primary antibodies (PCNA, ab92552, 1:500; KI67, ab16667, 1:500, both provided by Abcam) were diluted in 1% BSA and applied overnight at 4°C. After primary antibody incubation, sections were washed in phosphate‐buffered saline (PBS) and incubated with species‐specific biotinylated secondary antibodies (1:200, Vector Laboratories) for 1 h at room temperature. Detection was performed using an avidin‐biotin complex (ABC) reagent (Vectastain ABC Kit, Vector Laboratories) for 30 min, followed by color development with 3,3'‐diaminobenzidine (DAB; Vector Laboratories). Sections were counterstained with Mayer's hematoxylin (Sigma‐Aldrich), dehydrated, and mounted using DPX mounting medium. Slides were scanned using an Aperio AT2 digital slide scanner (Leica Biosystems) and analyzed using QuPath software (v.0.3.2). Quantitative analysis of staining intensity and the percentage of positive cells was performed using predefined thresholds for DAB intensity. For each section, three random fields were selected for analysis at 200× magnification, and the mean staining intensity (measured as optical density) and percentage of positive cells were recorded. Positive cell populations were normalized to the total number of cells within each field.

### 2.9 Immunofluorescence Staining

For immunofluorescence staining, paraffin sections were deparaffinized and rehydrated, followed by antigen retrieval using sodium citrate buffer (pH 6.0) heated to 95°C in a microwave for 15 min. Sections were blocked with 5% normal donkey serum (Jackson ImmunoResearch) and 0.1% Triton X‐100 in PBS for 1 h at room temperature. Primary antibodies were diluted in 1% BSA and incubated overnight at 4°C. The following primary antibodies were used: anti‐DESMIN (1:100, Abcam), anti‐SMA (1:200, Abcam), anti‐CD8 (1:200, BioLegend), and anti‐VEGFA (1:100, Abcam). The next day, sections were washed three times in PBS and incubated with species‐specific secondary antibodies conjugated to Alexa Fluor 488, 594, or 647 (Thermo Fisher Scientific, 1:500) for 1 h at room temperature in the dark. Nuclei were counterstained with 4',6‐diamidino‐2‐phenylindole (DAPI, 1 µg/mL, Thermo Fisher Scientific) for 5 min. After washing, slides were mounted with ProLong Gold antifade reagent (Thermo Fisher Scientific) and coverslipped. Immunofluorescence images were captured using a Nikon A1 confocal microscope, and Z‐stack images were acquired at 0.5 µm intervals. Co‐localization of DESMIN and SMA was assessed by calculating the Pearson correlation coefficient using NIS‐Elements software. Fluorescence intensity was quantified in three randomly selected fields per slide, and mean fluorescence intensity (MFI) was calculated for each marker using ImageJ.

For double‐label immunofluorescence, antibodies for DESMIN and SMA were applied sequentially, and secondary antibodies conjugated to different fluorophores were used for detection. Co‐localization of markers was visualized using the colocalization module in NIS‐Elements software, and the percentage of double‐positive cells was calculated by counting the number of co‐expressing cells relative to the total number of cells stained with each marker. Additionally, the MFI ratio of DESMIN to SMA was calculated to determine the relative expression levels of these markers in CAFs.

### 2.10 Flow Cytometry

For flow cytometric analysis, tumors, spleens, lungs, and liver tissues were harvested and placed in cold PBS with 2% FBS. Tumor tissues were mechanically dissociated and enzymatically digested with 2 mg/mL collagenase D, 0.5 mg/mL hyaluronidase, and 0.1 mg/mL DNase I for 30 min at 37°C. Lung and liver tissues were similarly digested for 20 min. After digestion, cell suspensions were filtered through a 70 µm strainer, centrifuged at 300 x g for 5 min, and resuspended in cold PBS with 2% FBS. Red blood cells were lysed using RBC lysis buffer and washed with PBS. After cell counting and viability assessment using Trypan Blue, 1–2 million cells were stained for surface markers. Cells were blocked with Fc receptor blocking solution (Anti‐Mouse CD16/CD32) for 10 min at 4°C, followed by incubation with fluorophore‐conjugated antibodies for TAMs (CD206, Ly6C, CD86), T cells (CD8, CD69, PD‐1, CTLA4), and macrophages (F4/80, CD11b, CD14, CD68) for 30 min at 4°C. After surface staining, cells were washed and fixed with BD Cytofix/Cytoperm for intracellular cytokine staining of IFN‐γ, Granzyme B, and FoxP3, using permeabilization buffer for 30 min. Samples were acquired on a BD LSRFortessa flow cytometer, and at least 1 00 000 events per sample were collected. Data were analyzed using FlowJo software, with gating applied to exclude dead cells and doublets. Immune cell subsets were identified based on marker expression, and the mean fluorescence intensity (MFI) was used to quantify protein levels.

### 2.11 RAW264.7 Or BMDM Co‐Culture

For co‐culture experiments, RAW264.7 macrophages were seeded at 1 × 10^5^ cells per well in 6‐well plates and allowed to adhere overnight in DMEM with 10% FBS. eGFP‐DESMIN^+^ CAFs were seeded at a 3:1 ratio on top of the macrophages. After 24 h of co‐culture, the medium was replaced with fresh DMEM containing rIL‐8 (10 ng/mL; PeproTech) or an CXCR2‐neutralizing antibody (SB225002, 1 µm; Sigma‐Aldrich) to assess the effects of IL‐8 signaling on TAM polarization. Co‐culture was continued for an additional 48 h. After co‐culture, macrophages were collected and stained for TAM markers (CD206^+^Ly6C^+^) using flow cytometry. Supernatants were collected and stored at −80°C for cytokine analysis by enzyme‐linked immunosorbent assay (ELISA) for IL‐8, VEGFA, and TGF‐β1 (R&D Systems).

To assess the impact of CAF‐derived IL‐8 on macrophage polarization, RAW264.7 cells were co‐cultured with CAFs for 48 h. For CXCR2 inhibition, SB225002 was added to the culture medium. Following co‐culture, RAW264.7 cells were harvested, and their polarization status was determined by flow cytometry using antibodies for M2 macrophage markers CD206 (APC‐conjugated, BioLegend) and Ly6C (FITC‐conjugated, BioLegend), and for M1 macrophage markers CD86 (PE‐conjugated, BioLegend).

### 2.12 Naïve T Cells Activation and Co‐Culture

Naïve CD8^+^ T cells were isolated from the spleens of 6‐ to 8‐week‐old B6 mice using a magnetic‐activated cell sorting (MACS) system. Briefly, spleens were harvested and mechanically dissociated by pressing through a 70 µm cell strainer (BD Falcon) to create a single‐cell suspension. The suspension was centrifuged at 300 *g* for 5 min, and red blood cells were lysed using an RBC lysis buffer (BioLegend) for 2 min at room temperature. The remaining splenocytes were washed with PBS containing 2% FBS and 1 mM EDTA (FACS buffer). Naive CD8^+^ T cells were then purified using a CD8a^+^ T Cell Isolation Kit (Miltenyi Biotec), following the manufacturer's instructions. The purity of isolated CD8^+^ T cells (> 95%) was confirmed by flow cytometry using anti‐CD8 (PE‐Cy7‐conjugated, BioLegend, Clone 53–6.7) and anti‐CD62L (APC‐conjugated, BioLegend, Clone MEL‐14) antibodies. Isolated naive CD8^+^ T cells were activated using anti‐CD3/CD28 Dynabeads (Thermo Fisher) at a bead‐to‐cell ratio of 1:1. Cells were cultured in RPMI‐1640 (Gibco) supplemented with 10% FBS, 1% penicillin‐streptomycin (Gibco), and 1% L‐glutamine (Sigma‐Aldrich). The cells were incubated at 37°C in a humidified atmosphere of 5% CO2 for 48 h to ensure activation. Activation was confirmed by flow cytometry, measuring the upregulation of activation markers CD69 (APC‐conjugated, BioLegend, Clone H1.2F3) and CD25 (FITC‐conjugated, BioLegend, Clone PC61).

For co‐culture experiments, activated CD8^+^ T cells were co‐cultured with macrophages in Transwell plates. RAW264.7 macrophages were pre‐polarized by co‐culture with cancer‐associated fibroblasts (CAFs) in the presence or absence of CXCR2 inhibitors for 48 h. The macrophages were then transferred to the lower chambers of Transwell plates (0.4 µm pore size, Corning), while activated CD8^+^ T cells were seeded in the upper chambers. Co‐culture was maintained in RPMI‐1640 medium for 72 h at 37°C and 5% CO_2_. To investigate the effect of IL8/IL8R signaling on T cell activation and exhaustion, the co‐culture was treated with either recombinant IL‐8 (10 ng/mL, PeproTech), the CXCR1 antagonist Reparixin (10 nm, MedChemExpress), or the CXCR2 antagonist SB225002 (1 µm, Sigma‐Aldrich). After 72 h of co‐culture, CD8^+^ T cells were collected from the upper chamber of the transwell plates and stained for surface markers of activation and exhaustion. To assess the cytotoxic function of CD8^+^ T cells, a cytotoxicity assay was performed after co‐culture. Target 3LL cells were labeled with carboxyfluorescein succinimidyl ester (CFSE, Thermo Fisher) and co‐cultured with CD8^+^ T cells at an effector‐to‐target ratio of 10:1 for 4 h at 37°C. After co‐incubation, target cell lysis was measured by staining with 7‐aminoactinomycin D (7‐AAD, BioLegend), and the percentage of dead (7‐AAD^+^) CFSE^+^ cells was determined using flow cytometry. The cytotoxic activity of CD8^+^ T cells was compared between different conditions (IL‐8‐treated, Reparixin‐treated, SB225002‐treated, and control).

### 2.13 WB Analysis

Tumor tissues or bone marrow were subjected to lysis using pre‐chilled RIPA buffer (50 mm Tris, pH 7.4, 150 mm NaCl, 1% nonidet P‐40, 0.5% sodium deoxycholate, and 0.1% sodium dodecyl sulfate), supplemented with a protease inhibitor cocktail (Roche) and a phosphatase inhibitor mixture (Thermo Scientific). The protein concentration in the cell lysates was quantified using a BCA kit (Thermo Fisher Scientific). For polyacrylamide gel electrophoresis, 20 µg of cell lysate from each sample was used. The proteins were transferred onto a polyvinylidene difluoride (PVDF) membrane, which was subsequently blocked with 5% non‐fat milk overnight. Following this, the membrane was incubated with primary antibodies at room temperature for 2 h. The membrane was washed thrice and then exposed to HRP‐conjugated anti‐mouse or anti‐rabbit secondary antibodies at a concentration of 1:5,000 at room temperature for 1.5 h. Subsequently, the membrane was washed again and exposed to chemiluminescent substrates (Western Lightning Plus ECL kit, PerkinElmer) before being visualized using X‐ray film for image analysis.

### 2.14 RNA Extraction and RT‐qPCR Analysis

Total RNA was extracted from these cells employing TRIzol (Ambion, Lot: 260808), followed by purification using the miRNeasy Mini kit (Qiagen, 157029493, Thermo Scientific). The quality and quantity of the RNA were assessed using a NanoDrop 2000 spectrophotometer. For cDNA synthesis, 1 µg of RNA was subjected to reverse transcription utilizing the qscriptXLT cDNA superMix reagent (Quantabio, 66141329). Subsequent to cDNA synthesis, quantitative PCR was conducted using the SYBR Green FastMix Low ROX reagent (TaKaRa) on a QuantStudio real‐time PCR system (ThermoFisher Scientific).

### 2.15 Statistical Analysis

All statistical analyses were performed using GraphPad Prism 9.0 (GraphPad Software, San Diego, CA, USA) and R software (version 4.4.0). Before analysis, data were evaluated for normality using the Shapiro–Wilk test and for homogeneity of variance using Levene's test. Outliers were identified by the Grubbs’ test and excluded where appropriate. Continuous data are presented as mean ± standard deviation (SD) unless otherwise specified. For comparisons between two groups, a two‐sided unpaired Student's *t*‐test was used for normally distributed data; otherwise, the Mann–Whitney U test was applied. For comparisons among three or more groups, one‐way ANOVA (with Tukey's multiple‐comparison post‐hoc test) or two‐way ANOVA (with Bonferroni correction) was performed depending on the experimental design. Survival analyses were conducted using the Kaplan–Meier method, with differences assessed by the log‐rank test. Correlation analyses were performed using Pearson's correlation coefficient for normally distributed variables or Spearman's rank correlation for nonparametric data. A two‐tailed alpha value of 0.05 was considered statistically significant. Sample sizes (n) for each experiment are indicated in the corresponding figure legends. Significance levels are represented as follows: *p < 0.05, **p < 0.01, ***p < 0.001, and ****p < 0.0001. All experiments were independently repeated at least three times, and representative data are shown.

## 3 Results

### 3.1 CAFs Facilitate Tumorigenesis and TAMs Accumulation in Aggressive Murine Tumor Models via the IL‐8/CXCR2 Axis

We first analyzed the expression levels of DESMIN in tumor tissues and adjacent normal tissues of 118 LC patients using TMA. We found that the expression of DESMIN was significantly higher in tumor tissues compared to adjacent normal tissues (Figure S1A). Additionally, double‐label immunofluorescence confirmed the co‐localization of DESMIN with SMA, indicating that DESMIN is primarily expressed in stromal cells (Figure S1B). Moreover, in the TCGA database, DESMIN expression levels showed a significant positive correlation with the expression levels of pan‐myeloid cell markers (CD33, CD14, and CD68) (Figure S1C). To further confirm the clinical relevance of these findings, we analyzed the correlation between DESMIN staining intensity and the expression of CD14, CD11b, and CD68 using TMA. High DESMIN expression was positively correlated with an extensive stromal reaction, characterized by dense collagen deposition and the accumulation of myeloid cells (CD14, CD11b, and CD68‐positive cells) (Figure S1D–E). We also found that the DESMIN‐high group was associated with a more advanced stage and increased lymph node metastasis compared to the DESMIN‐low group (Figure S1F–H). Furthermore, high DESMIN expression was independently associated with recurrence, positive lymph node metastasis, undifferentiated pathology, and microsatellite stability (MSS) status (Figure S1I). The DESMIN‐high group exhibited the lowest overall survival, disease‐specific survival, and recurrence‐free survival rates (Figure S1J–L). Considering the cellular complexity of the TME and the fact that multiple cell types can produce IL‐8, we quantified IL‐8 expression and secretion in DESMIN^+^ or DESMIN^−^ CAFs, mouse 3LL lung tumor cells, and mouse RAW264.7 macrophages. Among these, DESMIN^+^ CAFs showed the highest IL‐8 mRNA expression (Figure S1M,N). These findings support the notion that DESMIN^+^ CAFs are the major contributors to the elevated IL‐8 levels. Furthermore, DESMIN^+^ CAFs expressed higher levels of activated CAF markers, including FAP and α‐SMA (Figure S1O). This is consistent with our clinical observations that IL‐8 levels correlate specifically with CAF‐rich stroma.

Our recent research has demonstrated that CAFs and their secretory cytokines play a fundamental role in the formation of an immunosuppressive TME, and the association between the CAFs‐derived IL‐8/IL‐8R axis and TAM infiltration and tumor progression has been revealed [18]. To delve into this issue, we generated a murine model of LC by injecting the 3LL cells alone or the mixture of CAFs and 3LL cells (1:3). Notably, mice injected with the mixture showed substantially increased serum levels of IL‐8 compared to those injected with 3LL cells alone. Additionally, other chemokines secreted by CAFs, like CCL2, CCL5, and CXCL12, were also upregulated in the mixture injection group, though to a lesser extent than IL‐8 (Figure S2A). Notably, it was found that the tumor tissues formed by the cell mixture (CAFs‐3LL) showed significantly higher expression of IL‐8 and CXCR2 (Figure S2B,C). Moreover, increased expression of CXCR2 was also found in the bone marrow of mice implanted with the cell mixture, with a significantly increased population of CXCR2^+^CD206^+^Ly6C^+^ cells (Figure S2D,E). In addition, data from the TCGA‐LUAD database suggested that IL‐8 and CXCR2 were upregulated in patients expressing high levels of CAF markers fibronectin 1 (FN1) and vimentin (VIM) (Figure S2F,G). Furthermore, MTOs were prepared and injected into B6 mice orthotopically, followed by the treatment of CXCR2‐specific inhibitors SB225002 or Navarixin (Figure S2H). Notably, this inhibition of CXCR2 signaling significantly reduced the number of metastatic nodules in the lung and liver nodes (Figure S2I,J). Meanwhile, the number of M2‐like TAMs (CD206^+^Ly6C^+^) in both primary and metastatic tumors was significantly reduced following SB225002 or Navarixin treatment (Figure S2K,L).

**FIGURE 1:**
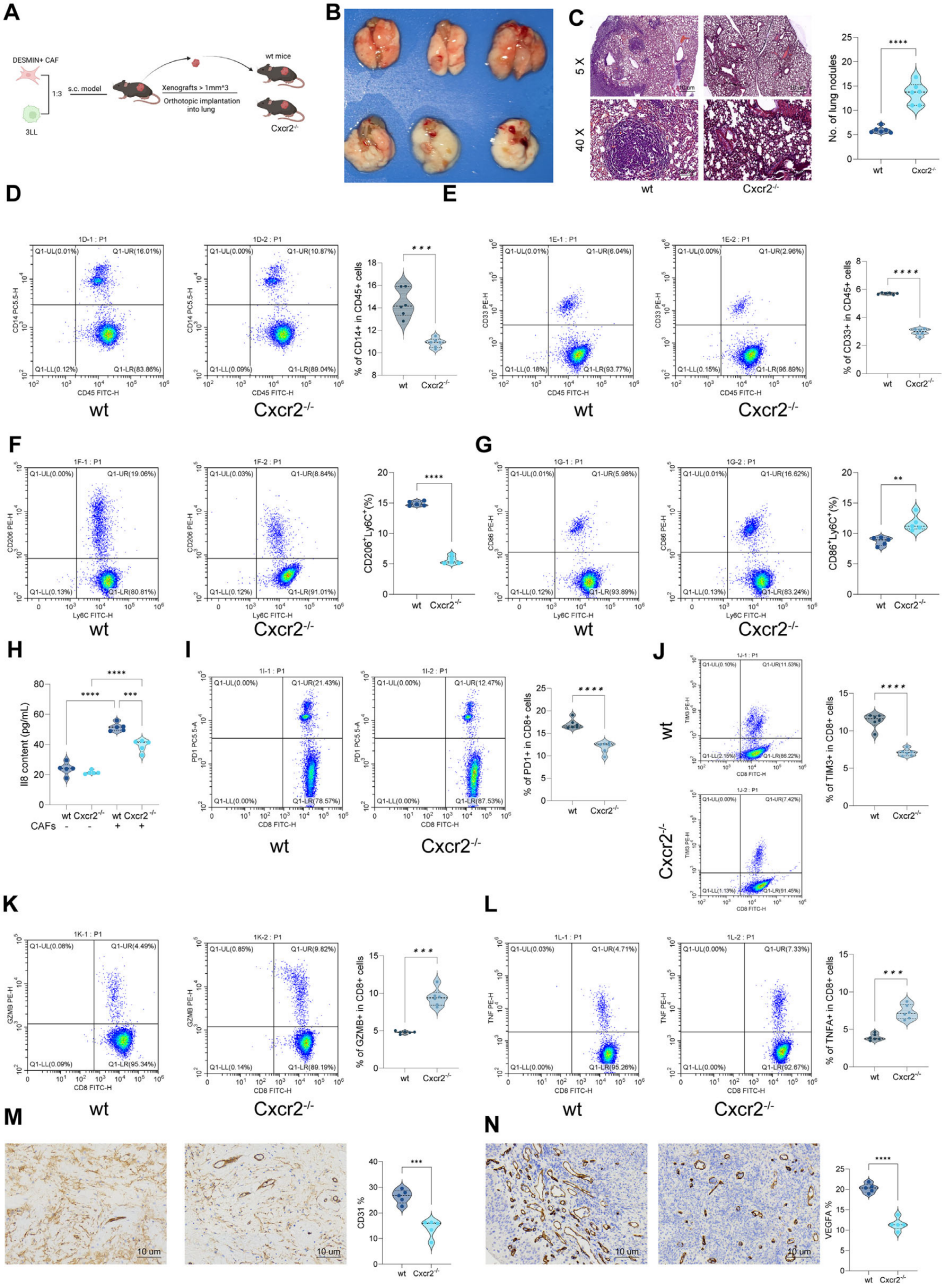
*Cxcr2* deletion reduces tumorigenesis and immune evasion in mice. (A) schematic illustration of the mouse treatment. The 3LL tumor cells were implanted alone or mixed with CAFs at a 3:1 ratio subcutaneously, and the tumors (MTOs) were transplanted into WT or *Cxcr2* ^−/−^ mice orthotopically. After 5 weeks, mice were euthanized, and tumor tissues were harvested for analyses. (B) representative images of tumor nodes in the mouse lung tissues; (C) number of nodules in the mouse lung tissue sections; (D, E) populations of CD45^+^CD14^+^ (D) and CD45^+^CD33^+^ (E) in the mouse lung tissue determined using flow cytometry; (F, G) populations of CD206^+^Ly6C^+^ (F) or CD86^+^Ly6C^+^ (G) cells in the mouse lung tumor tissues determined using flow cytometry; (H) serum levels of IL‐8 in mice determined using ELISA kits; (I–N) populations of PD‐1^+^ CD8^+^ T (I), TIM3^+^CD8^+^ T (J), GZMB^+^CD8^+^ T (K), and TNFA^+^ CD8^+^ T (L) cells in the orthotopic tumor tissues determined using flow cytometry. (M, N) positive staining of CD31 (M), and VEGFA (N) in the mouse lung tumor tissue sections determined using IHC.Each dot indicates one independent experiment. **p < 0.01, ***p < 0.001, ****p < 0.0001.

Furthermore, MTOs derived from 3LL cells or the mixture of 3LL cells and eGFP‐DESMIN CAFs (mixed at 3:1) were injected into WT or *Cxcr2* ^−/−^ mice subcutaneously (Figure 1A). Notably, it was observed that CAFs promoted the metastasis of 3LL cells to the mouse liver tissues (Figure S3A), but the tumor burden in the lung was reduced in *Cxcr2* ^−/−^ mice (Figure 1B,C). In addition, the *Cxcr2* deletion in mice significantly decreased the population of infiltrated myeloid cells in both primary lung tumors and metastatic nodules in the liver (Figure S3B,C) (Figure 1D,E).

To further examine the function of CAFs‐secreted IL‐8 in TAM differentiation, we had RAW264.7 macrophages co‐cultured with DESMIN^+^ CAFs in vitro, followed by the addition of IL‐8‐specific monoclonal antibody (anti‐IL‐8) (Figure S4A). The CAF stimulation significantly promoted the immunosuppressive polarization of TAMs and increased the levels of M2‐like TAM markers (CD206, CD36, and Arg1), which were markedly negated by the supplementation of anti‐IL‐8 (Figure S4B,C).

In another scenario, a CXCR2 neutralizing antibody (anti‐CXCR2) was added to the co‐culture system of RAW264.7 macrophages and DESMIN^+^ CAFs (Figure S4D). Still, co‐culturing with these CAFs significantly increased the expression of M2 TAM markers (CD206, CD36, and Arg1) while reducing the expression of M1 markers (CD86 and iNOS) (Figure S4E–G), accompanied by increased secretion of immunosuppressive cytokines TGFβ and CSF1 while decreased release of immunoreactive cytokines TNFα and IL‐6 (Figure S4H). The migration of these macrophages was increased upon co‐culturing with CAFs as well (Figure S4I). However, these effects were negated by the addition of anti‐CXCR2 to the co‐culture system (Figure S4E–I). Furthermore, a culture system of RAW264.7 macrophages was added with the mouse IL‐8 recombinant protein (rIL‐8) or the additional anti‐CXCR2 (Figure S4J). Importantly, the proportion of CD206^+^Ly6c^+^ (M2) cells was significantly increased by the rIL‐8 treatment but reduced by the administration of anti‐CXCR2 (Figure S4K). Meanwhile, the concentrations of M2 markers CSF1, TGFβ, and VEGFA were promoted by rIL‐8 but reduced by anti‐CXCR2 (Figure S4L).

In the mouse tumor models, it was found that the population of CD206^+^Ly6C^+^ (M2) cells was increased while the population of CD86^+^Ly6C^+^ (M1) cells was decreased in the CAF ^+^ 3LL group, effects diminished in *Cxcr2* ^−/−^ mice (Figure 1F,G) (Figure S3D,E). The IL‐8 level in the serum was significantly increased upon CAF implantation in both WT and *Cxcr2* ^−/−^ mice (Figure 1H). Moreover, flow cytometry analysis showed that the populations of exhausted CD8^+^ T cells (TIM3^+^/PD‐1^+^), in both lung tumors or metastatic nodules in the liver, were substantially reduced in the *Cxcr2* ^−/−^ mice. By contrast, the populations of effector/cytotoxic CD8^+^ T cells (TNFA^+^/GZMB^+^) were increased (Figure 1I–L) (Figure S3F–I). Similar trends were observed in the expression of angiogenesis markers CD31 and VEGFA (Figure 1M,N) (Figure S3J,K).

### 3.2 Knock‐Out of Cxcr2 in Mouse Myeloid Cells Reduces Tumorigenesis and Enhances Immune Activity

**FIGURE 2:**
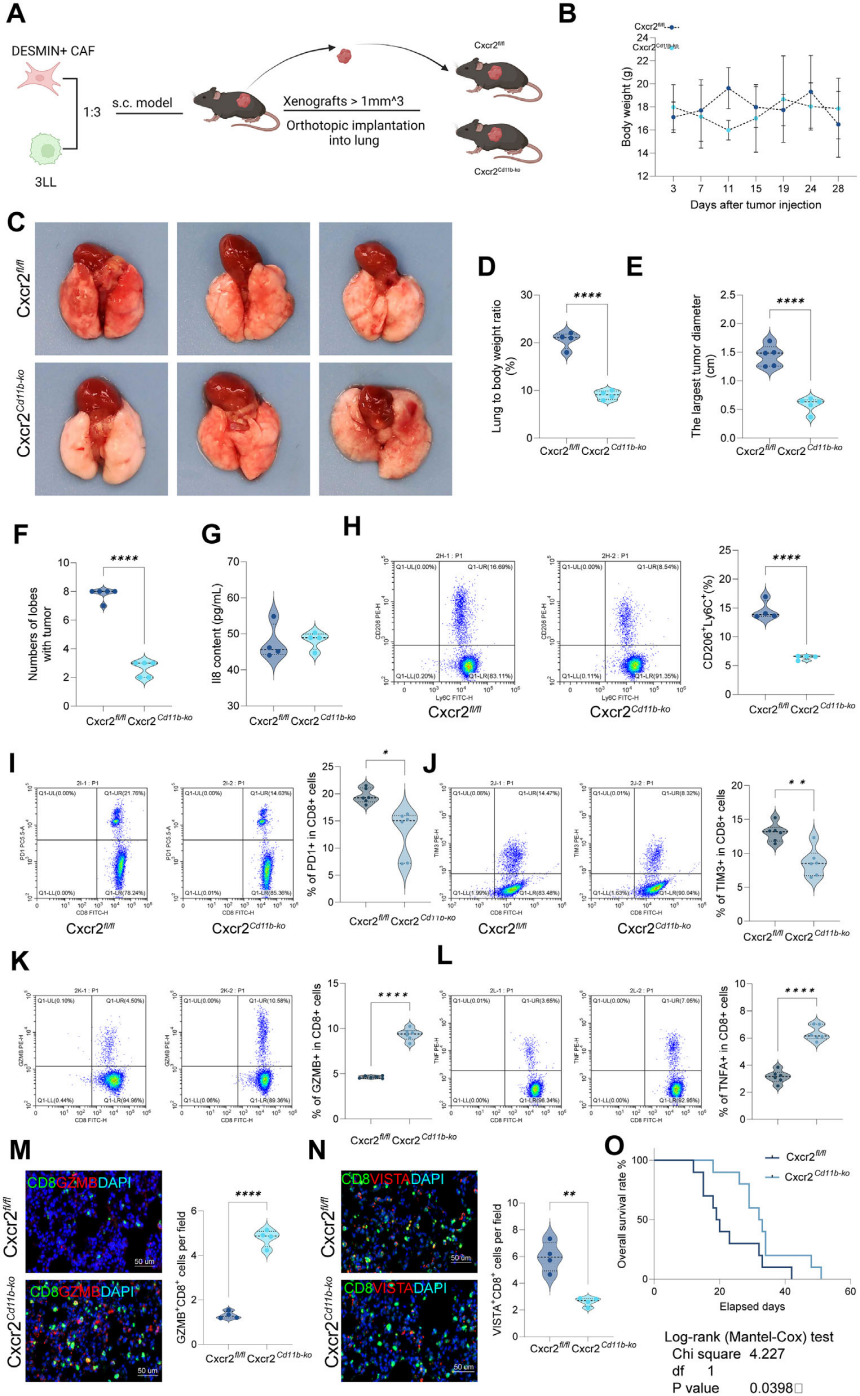
Knock‐out of *Cxcr2* in mouse myeloid cells reduces tumorigenesis and enhances immune activity. (A) Schematic illustration of the animal modeling: 3LL cells were inoculated subcutaneously in B6 mice along with DESMIN^+^ CAFs (3:1). After 4 weeks, the MTOs were orthotopically transplanted into the lung of *Cxcr2* ^fl/fl^ mice and the offspring of mice mated between CD11b‐Cre and *Cxcr2* ^fl/fl^ (*Cxcr2* ^CD11b‐KO^ mice); (B) the body weight of mice in each group after the traetment; (C–F) growth of MTOs in *Cxcr2* ^fl/fl^ and *Cxcr2* ^CD11b‐KO^ mice; (G) serum IL‐8 contents in *Cxcr2* ^fl/fl^ and *Cxcr2* ^CD11b‐KO^ mice; (H) population of CD206^+^Ly6C^+^ cells in the orthotopic tumor tissues determined using flow cytometry; (I–L) populations of PD‐1+ CD8+ T (I), TIM3+CD8+ T (J), GZMB+CD8+ T (K), and TNFA+ CD8+ T (L) cells in the orthotopic tumor tissues determined using flow cytometry; (M, N) populations of GZMB^+^CD8^+^ T (M) cells or VISTA^+^CD8^+^ T (N) cells in the orthotopic tumor tissues determined using immunofluorescence staining; (O) overall survival rate of mice. Each dot indicates one independent experiment. *p < 0.05, **p < 0.01, ***p < 0.001, ****p < 0.0001.

While IL‐8 has two receptors, CXCR1 and CXCR2 in human, mice do not have a functional CXCR1 receptor but only express CXCR2, which binds to IL‐8‐like chemokines. To confirm whether IL‐8 promotes immunosuppressive macrophage polarization through CXCR1 or CXCR2, we had PMA‐treated THP‐1 cells co‐cultured with IL‐8, followed by the addition of CXCR1 inhibitor Reparixin (which has higher affinity for CXCR1 than CXCR2) or the CXCR2 inhibitor SB225002. We observed that treatment with either Reparixin or SB225002 significantly suppressed immunosuppressive macrophage polarization; however, the effect of SB225002 was markedly stronger than that of Reparixin. These results indicate that IL‐8 primarily induces immunosuppressive macrophage polarization via CXCR2 (Figure 4M–O). Furthermore, the isograft tumors formed by a mixture of 3LL cells and DESMIN^+^ CAFs after 4 weeks (MTOs) were transplanted into the right upper lobes of the lungs of *Cxcr2* ^fl/fl^ mice, as well as the offspring of mice mated between CD11b‐Cre and *Cxcr2* ^fl/fl^ (which are referred to as *Cxcr2* ^CD11b‐KO^ mice) (Figure 2A). It was found that the growth of MTOs was significantly suppressed in *Cxcr2* ^CD11b‐KO^ mice (Figure 2B–F). The IL‐8 levels showed no significant differences between *Cxcr2* ^fl/fl^ and *Cxcr2* ^CD11b‐KO^ mice (Figure 2G), while the number of infiltrating CD206^+^Ly6C^+^ cells in the orthotopic and metastatic tumor tissues in *Cxcr2* ^CD11b‐KO^ mice was significantly decreased (Figure 2H) (Figure S5A). Additionally, the populations of PD‐1^+^ or TIM3^+^ CD8^+^ T cells were substantially reduced while the populations of GZMB^+^ or TNFA^+^ CD8^+^ T cells were increased in *Cxcr2* ^CD11b‐KO^ mice (Figure 2I–L) (Figure S5B–E). Notably, the number of T effector cells (GZMB^+^) in the primary or metastatic tissues in *Cxcr2* ^CD11b‐KO^ mice was significantly increased, whereas the number of dysfunctional T cells (VISTA^+^) was significantly decreased (Figure 2M,N) (Figure S5F,G). Moreover, it was observed that the survival period of *Cxcr2* ^CD11b‐KO^ mice significantly increased (Figure 2O). Subsequently, BMDMs from *Cxcr2* ^fl/fl^ mice were extracted. After being induced with M‐CSF and IL‐4, rIL‐8, and SB225002 were added, respectively (Figure S5H). It was observed that after rIL‐8 treatment, M2 polarization of myeloid cells was significantly increased; however, further treatment with SB225002 weakened the effect of rIL‐8 (Figure S5I,J). Additionally, the secretion of IL‐4, TGFβ, and VEGFA was significantly increased by rIL‐8, which was negated by the additional treatment of SB225002 (Figure S5K).

### 3.3 Loss of Cxcr2 in TAMs Improves Immune Activity of CD8+ T Cells

**FIGURE 3:**
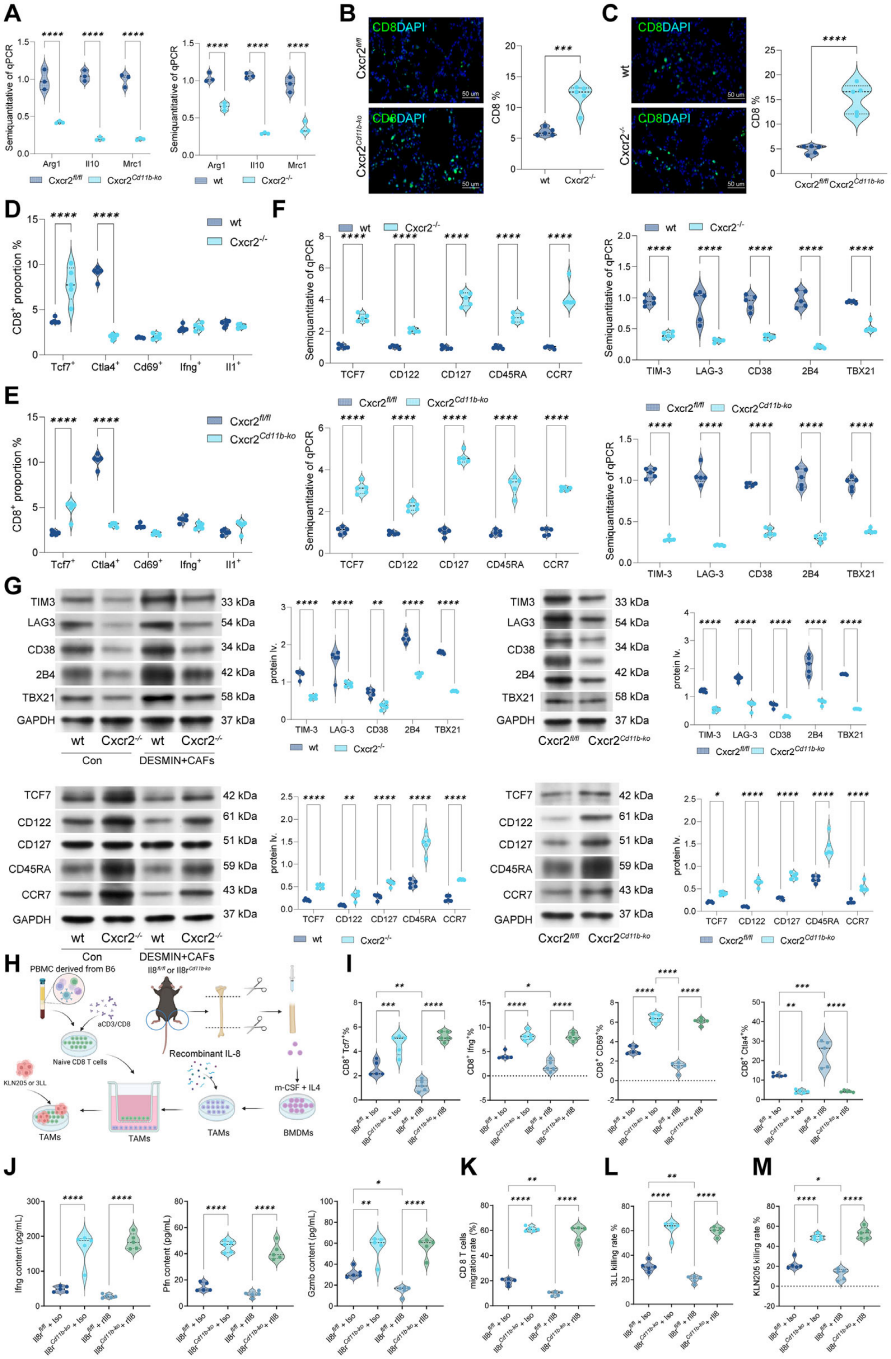
Loss of *Cxcr2* improves immune activity of CD8^+^ T cells. (A) mRNA expression of Arg1, IL‐10, and MRC1 in tumors from WT, *Il8* ^−/−^, *Cxcr2* ^fl/fl^, and *Cxcr2* ^CD11b‐KO^ mice determined using RT‐qPCR; (B, C) CD8^+^ T cells in tumors from each group of mice determined using immunofluorescence staining; (D, E) TCF7^+^, CTLA‐4^+^, CD69^+^, IFNγ^+^, and IL‐1^+^ CD8^+^T cells determined using flow cytometry; (F, G) mRNA and protein levels of CD8^+^ T cell dysfunction markers (TIM‐3, LAG‐3, CD38, 2B4, and TBX21) and stem‐like CD8 T cell signatures (TCF7, CD122, CD127, CD45RA, and CCR7) determined using RT‐qPCR and WB analysis; (H) schematic illustration of the collection and induction of mouse CD8^+^ T cells, TAMs, the co‐culture system of these two, and the addition of rIL‐8 or the isotype control; (I) proportions of CD69^+^, IFNγ^+^, TCF7^+^, and CTLA4^+^ CD8^+^T determined using flow cytometry; (J) concentrations of PFN, GZMB, and IFNG in the culture system determined using ELISA kits; (K) migration rate of CD8^+^ T cells after co‐culture determined using Transwell assays; (L, M) cytotoxicity of CD8^+^ T cells to 3LL (L) and KLN205 (M) cells. Each dot indicates one independent experiment. *p < 0.05, **p < 0.01, ***p < 0.001, ****p < 0.0001.

The findings above have demonstrated that the infiltration and M2 polarization of TAMs were significantly reduced in both *Cxcr2* ^−/−^ and *Cxcr2* ^CD11b‐KO^ mice bearing tumors. This was substantiated by the reduced mRNA expression of M2 markers Arg1, IL‐10, and MRC1 in the tumor tissues of *Cxcr2* ^−/−^ or *Cxcr2* ^CD11b‐KO^ mice compared to the WT or *Cxcr2* ^fl/fl^ mice (Figure 3A). It is well established that TAM infiltration can significantly suppress the activity and function of CD8^+^ T cells. Notably, we found that the number of CD8^+^ T cells in the tumor tissues formed by MTO in *Cxcr2* ^CD11b‐KO^ mice or in *Cxcr2* ^−/−^ mice was significantly increased (Figure 3B,C), with similar trends observed in liver metastases (Figure S6A,B). Further assessment of the CD8^+^ T cell population in these tumors showed that the number of stem‐like CD8^+^ T cells (TCF7^+^) significantly increased in *Cxcr2* ^−/−^ or *Cxcr2* ^CD11b‐KO^ mice, while the number of exhausted CD8^+^T cells (CTLA‐4) significantly decreased. However, no significant changes were observed in the number of proliferative T cells, IFNγ‐related, or IL‐1‐related T cells (Figure 3D,E) (Figure S6C,D). Additionally, it was found that the expression of dysfunction CD8^+^ T cell signatures (TIM‐3, LAG‐3, CD38, 2B4, and TBX21) was significantly decreased in *Cxcr2* ^−/−^ or *Cxcr2* ^CD11b‐KO^ conditions, while the expression of stem‐like CD8 T cell signatures (TCF7, CD122, CD127, CD45RA, and CCR7) was significantly increased (Figure 3F,G) (Figure S6E,F).

Subsequently, to further confirm how TAMs affected T cell population, naive T cells were extracted from the spleens of B6 mice, induced with anti‐CD3/CD28, and co‐cultured with macrophages from *Cxcr2* ^CD11b‐KO^ or *Cxcr2* ^fl/fl^ mice in a Transwell system (Figure 3H). It was observed that the T cell activation was significantly promoted when co‐culturing with TAMs derived from *Cxcr2* ^CD11b‐KO^ compared to those from *Cxcr2* ^fl/fl^ mice, with significantly increased proportions of CD69^+^, IFNγ^+^, and TCF7^+^ CD8^+^ T cells, while decreased numbers of CTLA4^+^CD8^+^T cells (Figure 3I). Meanwhile, the active proteins secreted by cytotoxic CD8^+^ T cells, including PFN, GZMB, and IFNG, were increased after co‐culture with TAMs derived from *Cxcr2* ^CD11b‐KO^ mice (Figure 3J). Meanwhile, the migration ability of these CD8^+^ T cells was increased under this condition as well (Figure 3K). After co‐culture with TAMs, these CD8^+^ T cells were extracted and co‐cultured with 3LL or KLN205 cells. Notably, it was observed that the cytotoxicity of CD8^+^ T cells was higher in the *Cxcr2* ^CD11b‐KO^ group (Figure 3L,M). However, the addition of rIL‐8 to the co‐culture system of *Cxcr2* ^CD11b‐KO^ TAM and T cells only slightly suppressed the activity and cytotoxicity of the CD8^+^ T cells (Figure 3I–M).

**FIGURE 4:**
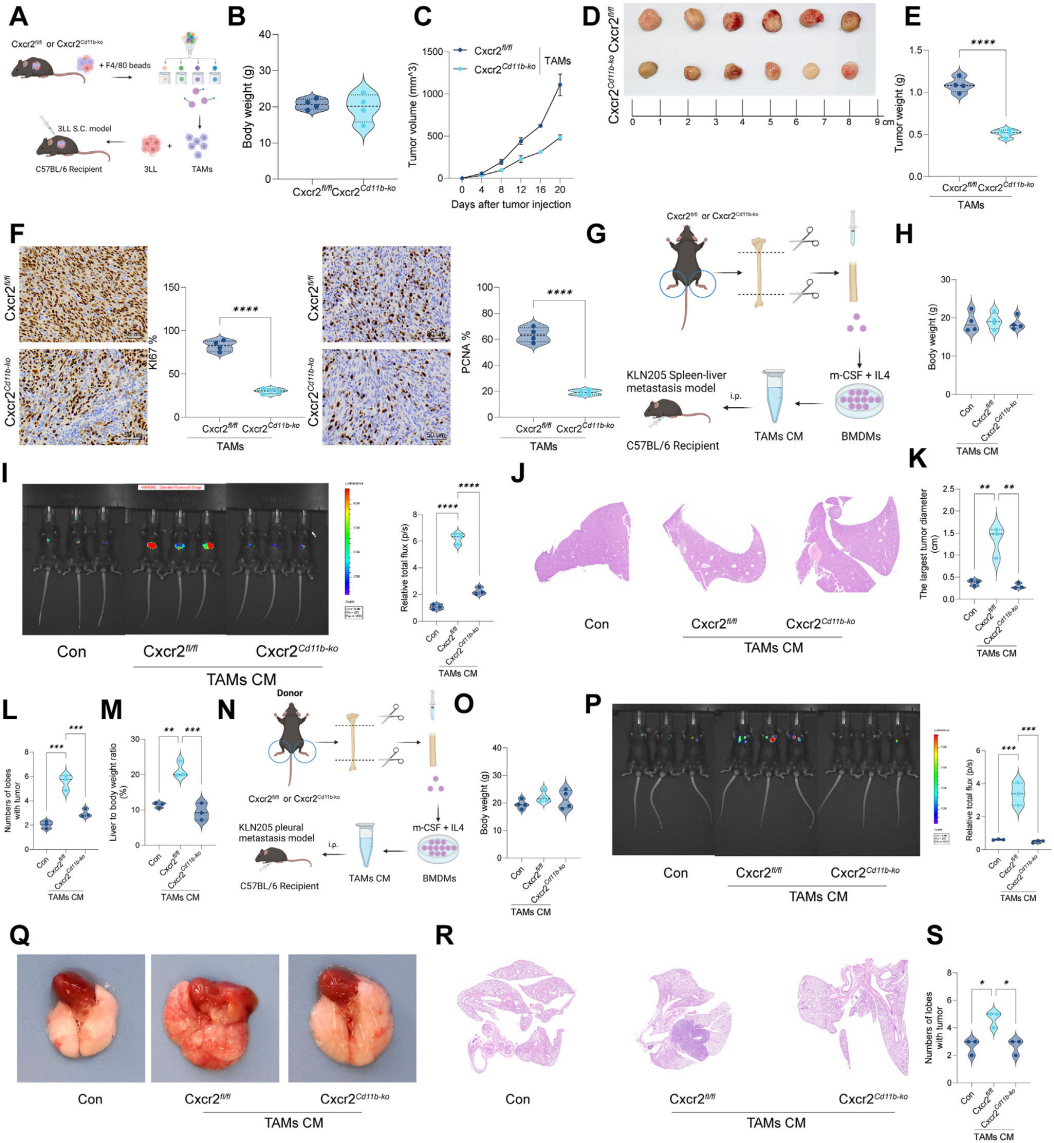
*Cxcr2* deletion in TAMs reduces tumor metastasis in mice. (A) schematic illustration of the modeling process; TAMs were isolated from 3LL‐derived tumors in *Cxcr2* ^fl/fl^ and *Cxcr2* ^CD11b‐KO^ mice using magnetic cell sorting, which were mixed with 3LL cells and injected into B6 recipient mice subcutaneously; (B) the body weight of mice in each group after the traetment; (C–E) volume and weight of the subcutaneous tumors in mice; (F) positive staining of Ki67^+^ or PCNA^+^ in the subcutaneous tumors determined using IHC; (G) schematic illustration of the modeling process; luciferase‐labeled KLN205 cells were administered along with TAM‐derived CM to B6 mice via splenic injection, and TAM‐derived CM was additionally intraperitoneally; (H) the body weight of mice in each group after the treatment; (I) representative bioluminescence images and statistical data of bioluminescence signal; (J) liver lobes examined by HE staining; (K) number of lobes with tumor; (L) the largest tumor diameter; (M) liver‐to‐body weight ratio; (N) schematic illustration of the modeling process; KLN205 cells were injected intrapleurally, while TAM‐derived CM was administered intra‐thoracically; (O) the body weight of mice in each group after the treatment; (P) representative bioluminescence images and statistical data of bioluminescence signal; (Q) representative gross lung images; (R) lobes determined using HE staining; (S) the number of lobes with metastatic nodules. Each dot indicates one independent experiment. *p < 0.05, **p < 0.01, ***p < 0.001, ****p < 0.0001.

The mouse 3LL cells were further injected into *Cxcr2* ^fl/fl^ and *Cxcr2* ^CD11b‐KO^ mice subcutaneously (Figure S6G). Compared to *Cxcr2* ^fl/fl^ mice, the growth rate of isograft tumors was slower in *Cxcr2* ^CD11b‐KO^ mice (Figure S6H–J), indicating that myeloid cells in the mice supported the in vivo growth of 3LL cells. Further investigation was conducted to validate whether the absence of *Cxcr2* in macrophages inhibited tumor progression. BMDMs from *Cxcr2* ^fl/fl^ or *Cxcr2* ^CD11b‐KO^ mice were collected and stimulated with IL‐4 and M‐CSF to induce an M2‐like TAM phenotype. These TAMs were mixed with 3LL cells and subcutaneously injected into B6 mice (Figure S6K). The deletion of *Cxcr2* in TAMs reduced the tumorigenic activity of 3LL cells (Figure S6L–N). Parallelly, TAMs were isolated from 3LL‐derived tumors in *Cxcr2* ^fl/fl^ and *Cxcr2* ^CD11b‐KO^ mice using magnetic cell sorting, which were mixed with 3LL cells and injected into B6 recipient mice subcutaneously (Figure 4A). The tumor growth rate of cancer cells mixed with *Cxcr2* ^CD11b‐KO^ TAMs was significantly slower than those mixed with *Cxcr2* ^fl/fl^ TAMs (Figure 4B–E). Meanwhile, tumors from the *Cxcr2* ^CD11b‐KO^ TAM group exhibited a lower proportion of Ki67^+^ or PCNA^+^ cells (Figure 4F).

TAMs typically interact with tumor cells or other immune cells in the TME by secreting various cytokines. Mice carrying 3LL‐cell derived tumors received intratumoral (i.t.) injections of conditioned media (CM) of TAMs derived from either *Cxcr2* ^fl/fl^ or *Cxcr2* ^CD11b‐ko^ mice (Figure S7A). Tumors injected with CM of *Cxcr2* ^CD11b‐KO^ TAMs showed slower growth compared to those injected with CM of *Cxcr2* ^fl/fl^ TAMs (Figure S7B–D). Parallel results were observed in the KLN205 cell subcutaneous injection model when using TAM‐derived CM for intratumoral injection (Figure S7E–H).

### 3.4 *Cxcr2* Deletion in TAMs Reduces Tumor Metastasis in Mice

To explore whether *Cxcr2* in TAMs participated in tumor metastasis, two LC metastasis models were established. First, in the spleen‐liver metastasis model, luciferase‐labeled KLN205 cells were administered along with TAM‐derived CM to B6 mice via splenic injection, while intraperitoneal injection of TAM‐derived CM was also performed (Figure 4G). Mice receiving CM of *Cxcr2* ^fl/fl^ TAMs displayed enhanced bioluminescence signals, liver/body weight ratios, tumor diameters, and the number of metastatic tumor nodules in the liver. These phenomena were reversed upon the knock‐out of *Cxcr2* in TAMs (Figure 4H–M). Second, in a KLN205 orthotopic lung metastasis model, highly metastatic KLN205 cells were injected intrapleurally, while TAM‐derived CM was administered intra‐thoracically (Figure 4N). The group treated with CM of *Cxcr2* ^CD11b‐KO^ TAMs exhibited fewer metastatic foci and smaller metastatic tumor diameters (Figure 4O–S).

Control mouse RAW 264.7 macrophages (referred to as RAW^Ctrl^) and RAW 264.7 cells overexpressing *Cxcr2* (referred to as RAW*^Cxcr2^* ^OE^) were stimulated with IFNγ and LPS to induce a tumor‐suppressive (M1) phenotype. The CM was collected, mixed with 3LL cells, and implanted into B6 mice subcutaneously (Figure S7I). It was found that ectopic expression of CXCR2 significantly increased the tumor growth in mice (Figure S7J‐M) and increased the proportions of Ki67^+^ and PCNA^+^ cells (Figure S7N). These data indicated that high levels of CXCR2 expression weakened the anti‐tumor phenotype of classical activated macrophages.

### 3.5 *Cxcr2*‐Expressing TAMs Disrupt T Cell‐Mediated Anti‐Tumor Immune Responses

**FIGURE 5:**
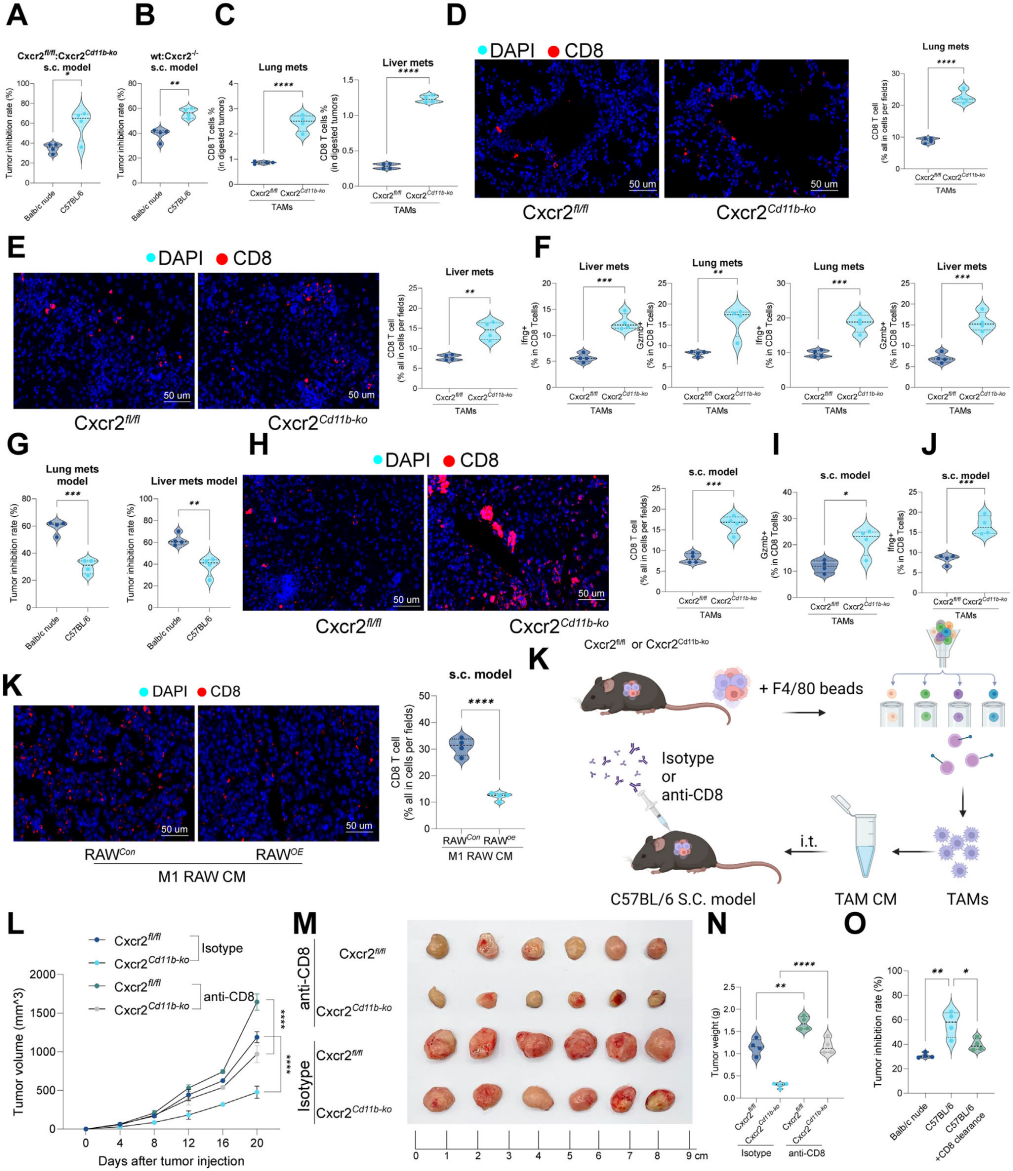
*Cxcr2* deletion in TAMs enhances T cell‐mediated anti‐tumor immune responses. (A, B) tumor inhibition rate in T cell‐deficient BALB/c nude mice and immunocompetent B6 mice bearing subcutaneous tumors, respectively; (C) CD8^+^ T cells in the lung and liver metastatic tumors in B6 mice determined using flow cytometry; (D, E) positive staining of CD8^+^ in the lung and liver metastatic tumors in B6 mice determined using immunofluorescence staining; (F) GZMB^+^ or IFNG^+^CD8^+^ T cells lung and liver metastatic tumors in B6 mice determined using flow cytometry; (G) tumor inhibition rate in B6 or BALB/c mouse models of metastatic tumors; H, CD8 T cells in the B6 subcutaneous tumor model determined using immunofluorescence staining; (I–J) GZMB^+^ or IFNG^+^CD8^+^ T cells in the B6 subcutaneous tumor model determined using flow cytometry; (K) proportion of CD8^+^ T cells subcutaneous tumors formed by the mixture of 3LL cells and the CM of RAW264.7 cells; (L) schematic illustration of the modeling process; systemic CD8^+^ T cell depletion was performed using anti‐CD8 antibodies in the KLN205 subcutaneous tumor model; (M–O) tumor volume and weight in the KLN205 subcutaneous tumor model with CD8^+^ T cell clearance; (O) tumor inhibition rate in BALB/c mice, B6 mice, or B6 mice with CD8^+^ T cell clearance. Each dot indicates data from one independent experiment. *p < 0.05, **p < 0.01, ***p < 0.001, ****p < 0.0001.

To further assess the immunosuppressive functions of *Cxcr2*‐expressing TAMs, a mixture of 3LL cells and TAMs, as depicted in Figure 4A, was injected into T cell‐deficient BALB/c nude mice (Figure S8A). In comparison to the 3LL cells mixed with *Cxcr2* ^fl/fl^ TAMs, the 3LL cells mixed with *Cxcr2* ^CD11b‐KO^ TAMs demonstrated lower tumor growth rates (Figure S8B–D). The tumor suppression rate in BALB/c nude mice was approximately 40% (Figure 5A), whereas it reached approximately 60% in B6 mice (Figure 5B). This finding strengthened the notion that *Cxcr2*‐expressing TAMs might disrupt T cell‐mediated anti‐tumor immune responses. In B6 mice, the loss of *Cxcr2* in TAMs increased CD8^+^ T cell infiltration in metastatic tumors in the lung and liver (Figure 5C,D and Figure S6A,B). Moreover, the absence of *Cxcr2* in TAMs elevated the expression of IFNγ and GZMB in infiltrating CD8^+^ T cells (Figure 5E). The role of CXCR2 in anti‐tumor immune responses was further assessed using a spleen‐liver metastasis model in BALB/c nude mice (Figure S8E). Compared to CM of *Cxcr2* ^fl/fl^ TAMs, the CM from *Cxcr2* ^CD11b‐KO^ TAMs mitigated bioluminescence signals, liver/body weight ratios, and tumor diameters in BALB/c nude mice (Figure S8F–I). However, the tumor suppression rate induced by the absence of *Cxcr2* in TAMs dropped from approximately 70% in immunocompetent mice to about 40% in immunodeficient mice (Figure 5F), confirming the regulatory role of *Cxcr2*‐expressing TAMs in T cell‐mediated anti‐tumor immunity. In addition, treatment with CM of *Cxcr2* ^fl/fl^ TAMs reduced CD8^+^ T cell infiltration in the metastatic tumors in the liver or lung, while these phenomena were reversed by *Cxcr2* knock‐out in TAMs (Figure 5G–I). Additionally, treatment with CM from M1‐like RAW 264.7 cells (as mentioned in Figure S7I) increased CD8^+^ T cell infiltration in tumors, while overexpression of *Cxcr2* abolished this effect (Figure 5J). To verify the involvement of CD8^+^ T cells in the anti‐tumor effects mediated by *Cxcr2* knock‐out, systemic CD8^+^ T cell depletion was performed using anti‐CD8 antibodies in the KLN205 subcutaneous tumor model (Figure 5K). Compared to the *Cxcr2* ^fl/fl^ group, treatment of CM of *Cxcr2* ^CD11b‐KO^ TAMs reduced tumor growth. However, this effect was partially diminished upon CD8^+^ T cell depletion (Figure 5L–N), with tumor suppression rates decreasing from ∼85% (IgG treatment group) to 35% (anti‐CD8) (Figure 5O).

### 3.6 The PI3K/AKT/RhoA/MRTF‐A/SRF Signaling Pathway Is Responsible for *CXCR2*‐Triggered M2 Polarization of TAMs

**FIGURE 6:**
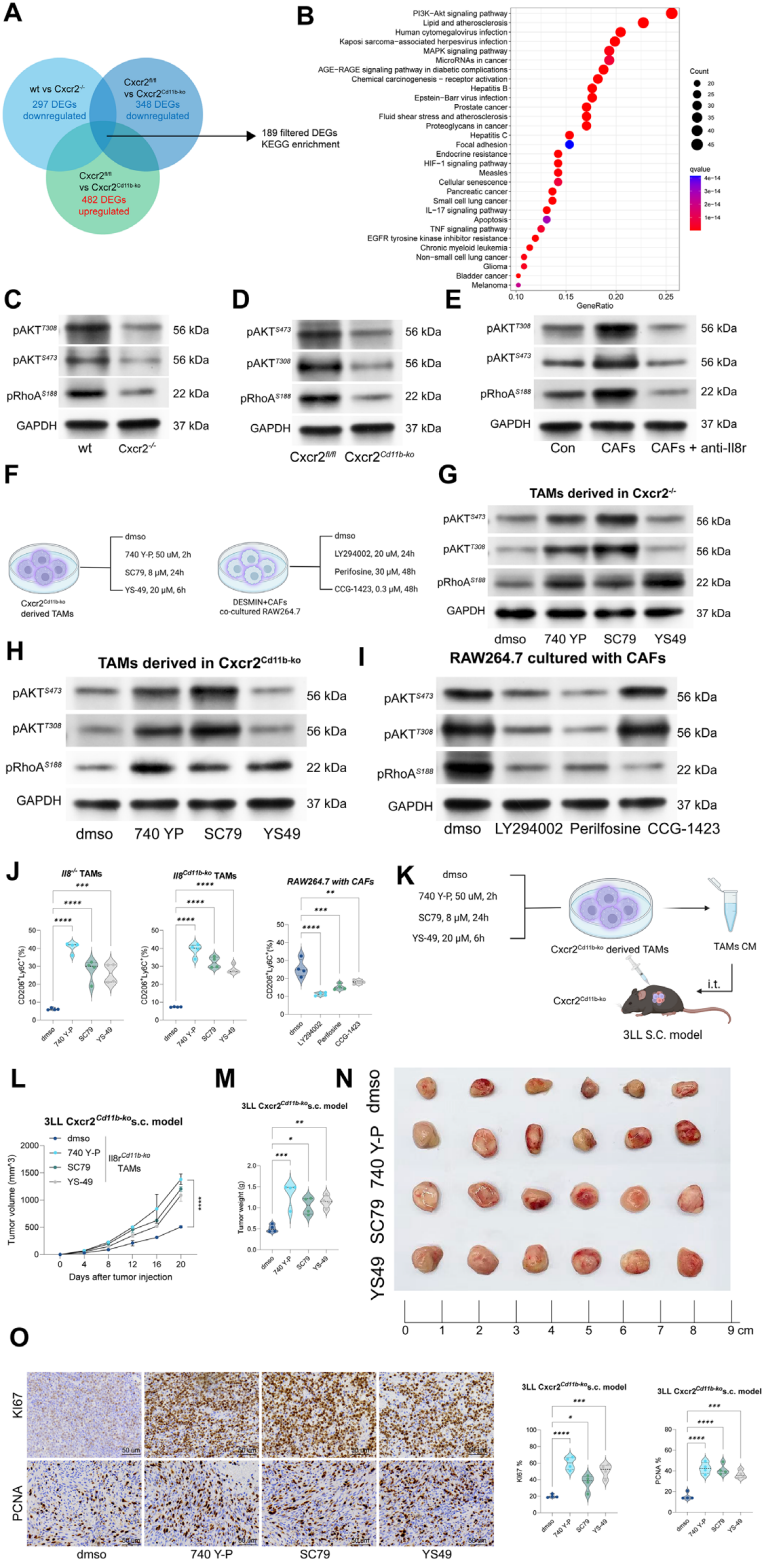
The PI3K‐AKT‐RhoA signaling pathway is responsible for CXCR2‐triggered M2 polarization of TAMs. (A) DEGs between BMDMs with or without systemic *Cxcr2* knock‐out, *Cxcr2* ^fl/fl^ or *Cxcr2* ^CD11b‐KO^ TAMs, and RAW264.7 macrophages co‐cultured with CAFs or not determined using RNA sequencing analyses; (B): KEGG enrichment analysis of the DEGs; (C–E): activation of pAKT (S473 and T308) and pRhoA (S188) in different macrophage models determined using WB analysis; (F) schematic illustration of in vitro experiments; TAMs derived from *Cxcr2* ^−/−^ or *Cxcr2* ^CD11b‐KO^ mice were treated with PI3K‐AKT‐RhoA agonists (740 Y‐P, SC79, and YS‐49), while CAF‐co‐cultured macrophages were treated with inhibitors (LY294002, Perifosine, and CCG‐1423) of the PI3K‐AKT‐RhoA pathway; (G–I) activation of pAKT (S473 and T308) and pRhoA (S188) in macrophages determined using WB analysis; (J) population of CD206^+^Ly6C^+^ macrophages determined using flow cytometry; (K) schematic illustration of the modeling process; the CM of TAMs derived from *Cxcr2* ^CD11b‐KO^ mice was injected intratumorally into *Cxcr2* ^CD11b‐KO^ mice bearing subcutaneous models; (L–N) tumor volume and weight in mice; (O) positive staining of KI67 and PCNA within the tumor tissues. Each dot indicates data from one independent experiment. *p < 0.05, **p < 0.01, ***p < 0.001, ****p < 0.0001.

To investigate the signaling pathways involved in the IL‐8/CXCR2‐mediated activation of TAMs, we performed RNA‐seq analysis on differentially expressed genes (DEGs) between BMDMs with and without systemic *Cxcr2* knock‐out, *Cxcr2* ^fl/fl^ or *Cxcr2* ^CD11b‐KO^ TAMs, and RAW264.7 macrophages co‐cultured with and without CAFs (Figure 6A). We identified a total of 189 intersecting genes, and KEGG enrichment analysis indicated that these genes are primarily involved in Chemokines‐Chemokine interaction and the PI3K/AKT/RhoA signaling pathways (Figure 6B).

Subsequently, WB analysis showed that in *Cxcr2* ^−/−^ or *Cxcr2* ^CD11b‐KO^ macrophages, levels of pAKT (S473 and T308) and pRhoA (S188) were lower compared to WT or *Cxcr2* ^fl/fl^ macrophages. Moreover, in macrophages co‐cultured with CAFs, the expression of pAKT (S473 and T308) and pRhoA (S188) was significantly elevated; however, this activation level decreased substantially when treated with an *Cxcr2* antibody (Figure 6C–E).

**FIGURE 7:**
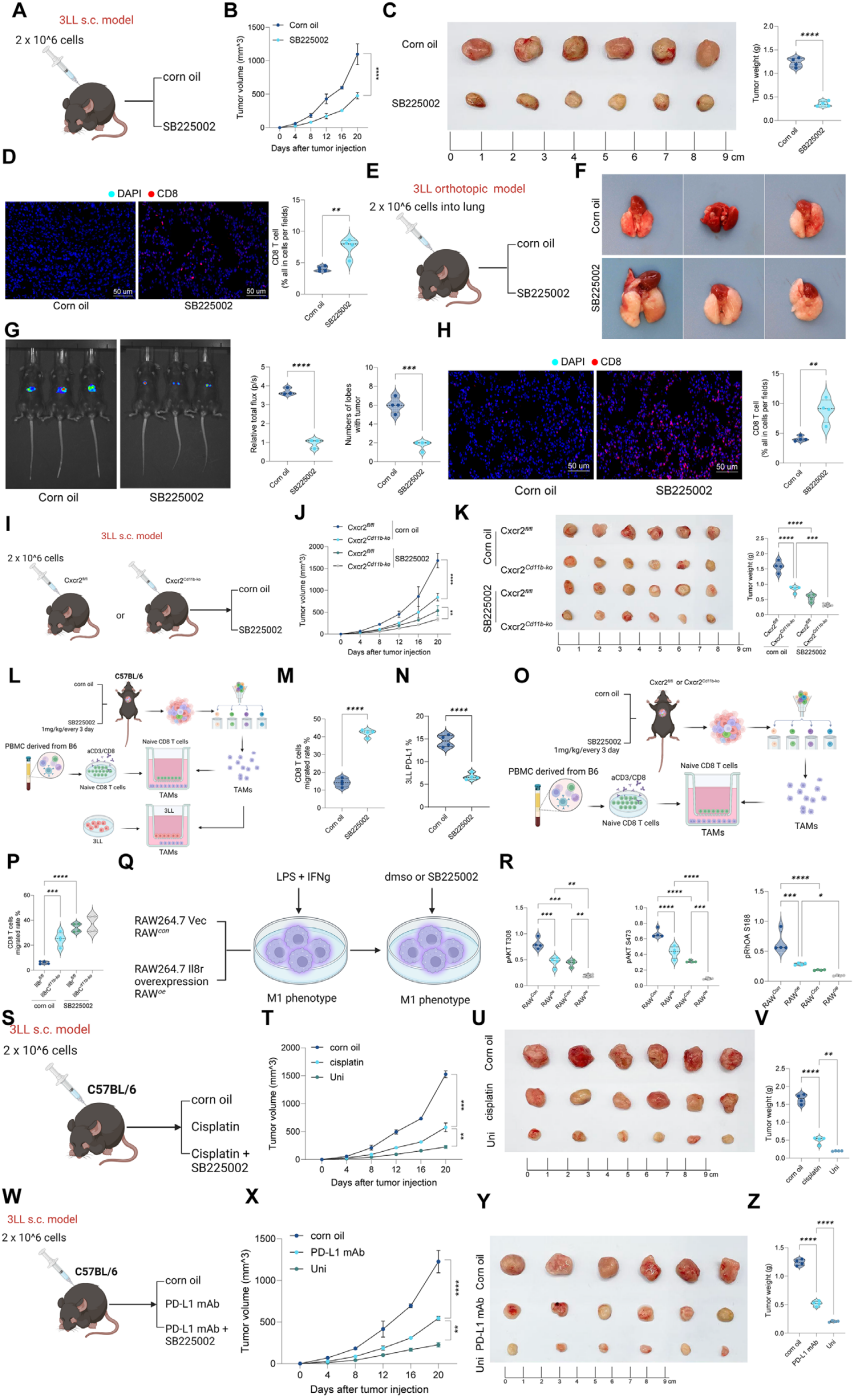
Blocking the IL‐8/CXCR2 interaction inhibits tumor growth and enhances immune response in mice. (A) schematic illustration of animal treatment; 3LL cells were injected into B6 mice subcutaneously, followed by treatment with corn oil or SB225002; (B, C) tumor volume and weight in mice; (D) positive staining of CD8 in tumor tissues determined using immunofluorescence staining; (E) schematic illustration of animal treatment; 3LL cells were injected into B6 mice orthotopically, followed by treatment with corn oil or SB225002; (F) representative gross lung images and bioluminescence images; (G) number of lobes with tumor; (H) positive staining of CD8 in tumor tissues determined using immunofluorescence staining; (I) schematic illustration of animal treatment; 3LL cells were injected into *Cxcr2* ^−/−^ or *Cxcr2* ^CD11b‐KO^ mice subcutaneously, followed by treatment with corn oil or SB225002; (J–K) tumor volume and weight in mice; (L) schematic illustration of experimental setting, TAMs were extracted from differentially treated B6 mice and co‐cultured with CD8 T cells or 3LL cells; (M) migration rate of CD8^+^ T cells determined using Transwell assays; (N) PD‐L1 expression in 3LL cells after co‐culture with TAMs determined using flow cytometry; (O) schematic illustration of experimental setting; TAMs were extracted from *Cxcr2* ^fl/fl^ or *Cxcr2* ^CD11b‐KO^ mice underwent corn oil or SB225002 treatment, which were co‐cultured with naïve CD8^+^ T cells derived from B6 mice; (P) migration rate of CD8^+^ T cells determined using Transwell assays; (Q) schematic illustration of experimental setting; RAW264.7 macrophages overexpressing *Cxcr2* or not were subjected to LPS and IFNG stimulation and received the additional treatment of SB225002 or DMSO; (R) activation of pAKT (S473 and T308) and pRhoA (S188) in macrophage models determined using WB analysis; (S) schematic illustration of experimental setting, B6 mice bearing 3LL subcutaneous models were treated with cisplatin treatment alone or the combination of cisplatin and SB225002; (T–V) tumor volume and weight; (W) schematic illustration of experimental setting, B6 mice bearing 3LL subcutaneous models were treated with PD‐L1 mAb alone or the combination of PD‐L1 mAb and SB225002; (X–Z) tumor volume and weight. Each dot indicates data from one independent experiment. **p < 0.01, ***p < 0.001, ****p < 0.0001.

We further utilized specific PI3K/AKT/RhoA agonists (740 Y‐P, SC79, and YS‐49) on TAMs derived from *Cxcr2* ^−/−^ or *Cxcr2* ^CD11b‐KO^ mice, while treating the CAF‐co‐cultured macrophages with specific inhibitors (LY294002, Perifosine, and CCG‐1423) of the PI3K/AKT/RhoA pathway (Figure 6F). For *Cxcr2* ^−/−^ or *Cxcr2* ^CD11b‐KO^ macrophages, activating the PI3K/AKT/RhoA pathway significantly promoted M2 TAM polarization (Figures 6G,H, 7J). Conversely, this was reversed in macrophages treated with antagonists (Figure 6I,J).

Next, we injected CM of *Cxcr2* ^CD11b‐KO^ derived TAMs into *Cxcr2* ^CD11b‐KO^ mice intratumorally (Figure 6K). The results showed that upon activation of the PI3K/AKT/RhoA pathway, the tumor growth rate in mice significantly increased, and there was a noticeable increase in the number of Ki67^+^ and PCNA^+^ cells (Figure 6L–O).

To further elucidate the downstream mechanism by which the IL‐8/IL‐8R‐mediated PI3K/AKT/RhoA pathway induces M2‐like polarization of TAMs, we examined myocardin‑related transcription factor A (MRTF‐A), a key downstream effector of RhoA. In macrophages treated with rIL‐8 or co‐cultured with CAFs, MRTF‐A was predominantly localized in the nucleus and exhibited increased binding to SRF, with reduced cytoplasmic retention (Figure S9A,B).

We hypothesized that activated RhoA promotes the polymerization of monomeric G‐actin into filamentous F‐actin. Under resting conditions, MRTF‐A is sequestered in the cytoplasm through binding to G‐actin; upon RhoA activation and G‐actin depletion (due to F‐actin formation), MRTF‐A is released and rapidly translocates to the nucleus. To test this, we performed ChIP‐seq using anti‐SRF antibodies. In rIL‐8‐treated macrophages, SRF binding to the genome was significantly increased (Figure S9C,D), with notable enrichment at the promoter regions of the M2‐associated genes Arg1 and Mrc1 (Figure S9E). These results indicate that CAF‐derived IL‐8 induces nuclear translocation of MRTF‐A in TAMs, which cooperates with SRF to transcriptionally activate M2 polarization genes. Consistent with this mechanism, the MRTF‐A‐specific small‐molecule inhibitor CCG‐1423 effectively reversed the IL‐8‐induced promotion of immunosuppressive M2‐like polarization in TAMs (Figs S9F–H).

### 3.7 Blocking the IL‐8/CXCR2 Interaction Inhibits Tumor Growth and Enhances Immune Response in Mice

To further confirm the role of IL‐8/CXCR2 in the progression of LC, we treated B6 mice bearing subcutaneous tumors with SB225002 (Figure 7A). This procedure significantly suppressed the volume and weight of tumors while increasing the number of CD8^+^ T cells within the tumor mass (Figure 7B–D). Additionally, in the in‐situ tumor model formed by 3LL cells, SB225002 also demonstrated effectiveness in inhibiting tumor progression (Figure 7E–H). SB225002 was further administered in the *Cxcr2* ^fl/fl^ and *Cxcr2* ^CD11b‐KO^ mouse tumor models (Figure 7I). The *Cxcr2* ^CD11b‐KO^ mice already presented reduced tumor growth than *Cxcr2* ^fl/fl^, application of SB225002 further suppressed tumor growth in both models (Figure 7J,K). Subsequently, TAMs were extracted from B6 mice and co‐cultured non‐contact with CD8 T cells or 3LL cells (Figure 7L). The results indicated that TAM extracted from mice treated with SB225002 significantly promoted the migration of CD8 T cells and inhibited the expression of PD‐L1 in 3LL cells (Figure 7M,N). Moreover, TAMs from *Cxcr2* ^fl/fl^ and *Cxcr2* ^CD11b‐KO^ mice were extracted as well and co‐cultured with of CD8 T cells (Figure 7O). While TAMs extracted from *Cxcr2* ^CD11b‐KO^ mice promoted migration of CD8^+^ T cells compared to those extracted from *Cxcr2* ^fl/fl^ mice, the administration of SB225002 no longer enhanced this effect (Figure 7P). Furthermore, in in vitro experiments, it was found that after treating RAW264.7 cells overexpressing *Cxcr2* with SB225002, the activation of the PI3K/AKT/RhoA pathway was significantly suppressed (Figure 7Q,R).

To further evaluate the effectiveness of interrupting the IL‐8/CXCR2 interaction in the treatment of LC, we treated mice bearing 3LL tumors with cisplatin alone or a combination of cisplatin and SB225002. The combined treatment significantly inhibited the growth of 3LL cells in vivo (Figure 7S–V). Similarly, the combination of SB225002 with PD‐L1 mAb presented more pronounced tumor‐inhibitory effect than the application of PD‐L1 mAb alone (Figure 7W–Z).

To further validate that DESMIN^+^ CAFs promote M2‐like polarization of macrophages and impair CD8^+^ T cell activity by remodeling the tumor microenvironment, we established PDOs from surgically resected lung cancer tissues. PDOs were co‐cultured with patient‐matched DESMIN^+^ CAFs and PBMCs in 3D Matrigel. SB225002 was added to the co‐culture system, followed by the introduction of PBMC‐derived CD8^+^ T cells (Figure S10A).

Addition of DESMIN^+^ CAFs markedly increased immunosuppressive M2‐like polarization of macrophages and promoted CD8^+^ T cell exhaustion. In contrast, treatment with SB225002 significantly attenuated M2‐like polarization (Figure S10B–D). Furthermore, DESMIN^+^ CAFs induced robust M2‐like polarization in human macrophages, accompanied by nuclear translocation of MRTF‐A (Figure S10E).

## 4 Discussion

Gaining insight into the cellular dynamics and interactions within the TME, as well as their links to disease progression and patient outcomes, is essential for therapy development, and advancements in precision oncology. Building upon our prior research, this study demonstrates that DESMIN^+^ CAFs promote LC growth and metastasis through the activation of the IL‐8/CXCR2 axis, with new mechanistic and translational insights.

The stromal component plays a pivotal role in the advancement of lung cancer and influences treatment outcomes [34]. Robust correlations exist between specific CAF populations and aggressive disease features. For instance, GPR77^+^ and CD10^+^ CAFs have been linked to factors such as tumor stemness and resistance to chemotherapy [15], while LRRC15^+^ CAFs exhibit tumor‐promoting and immunosuppressive properties [35]. A recent investigation by Chen et al. indicated that POSTN^+^ CAFs are more prevalent in advanced tumors, correlating with extracellular matrix remodeling, pathways of tumor invasion, and immune suppression [36]. Our previous study showed that tumor‐derived lactate drives DESMIN expression in CAFs, activating them and facilitating LC progression [18]. The present work extends these findings by demonstrating that DESMIN^+^ CAFs are the predominant source of IL‐8 in our models and act as key drivers of immunosuppression. While serum DESMIN levels have been proposed as a reliable prognostic indicator and therapeutic target in colorectal cancer [37], its prognostic relevance in various cancers, including lung cancer, remains largely uncharted. In this study, we identified DESMIN predominantly localized in stromal cells within clinical lung tumor specimens. High DESMIN expression correlated with increased recurrence, lymph node metastasis, poor histological differentiation, and inferior survival, positioning DESMIN as a marker of activated CAFs and a negative prognostic indicator in LC. Supporting this, DESMIN^+^ CAFs expressed higher levels of activated CAF markers (FAP and α‐SMA) and secreted substantially more IL‐8 than DESMIN^−^ CAFs, 3LL tumor cells, or macrophages. These observations align with clinical data showing that IL‐8 levels correlate specifically with CAF‐rich stroma.

Our in vivo models revealed that co‐implantation of CAFs with 3LL cells elevated serum and tumor IL‐8 levels, CXCR2 expression, and M2‐like TAM accumulation compared with 3LL cells alone, providing initial evidence implicating the IL‐8/CXCR2 axis in CAF‐mediated tumor progression. IL‐8 (CXCL8), part of the CXC glutamic acid‐leucine‐arginine motif family, acts on CXC receptors to attract neutrophils and various myeloid cells [38]. It is often upregulated in malignant human tissues, with higher circulating levels observed in patients at advanced stages [39]. Within the TME, IL‐8 can be secreted by cancer cells, myeloid cells, and CAFs [40]. Prior research established that tumor‐derived IL‐8 may influence immune cell composition within the TME in a paracrine manner, or engage in autocrine signaling to facilitate oncogenic processes [41]. The chemokine IL‐8 has been shown to support tumoral progress and metastasis through various mechanisms, notably by nurturing the maintenance of cancer stem cells and promoting angiogenesis. Its ability to attract and modulate neutrophils and macrophages is particularly crucial [31, 38]. Notably, elevated IL‐8 levels in circulation among melanoma and NSCLC patients correspond with poor responses to anti‐PD‐1 therapies [42]. CAF‐derived IL‐8 has been found to induce the differentiation of myeloid cells into suppressor types or M2‐like TAMs, aiding in tumor immune evasion [43]. Although inhibiting IL‐8/CXCR2 signaling using neutralizing antibodies or small molecules has been shown to decrease malignancy and stemness in solid tumors [44, 45], its effects on TAM infiltration and polarization are less well‐studied. We discovered that treating RAW264.7 cells with rIL‐8 significantly curtailed their chemotactic migration and M2 polarization in vitro. Consistent with these insights, we observed that either pharmacological inhibition of CXCR2 (SB225002 or Navarixin) or genetic deletion of *Cxcr2* (global or myeloid‐specific) reduced M2‐like TAM polarization, restored CD8^+^ T cell effector function, suppressed tumor growth and metastasis, and prolonged survival. These results strongly implicate the IL‐8/CXCR2 pathway in the immunosuppressive effects mediated by DESMIN^+^ CAFs.

The immune‐reactivating and tumor suppressive properties upon IL‐8/CXCR2 blockade were partially T cell‐dependent, as evidenced by reduced efficacy in immunodeficient models and CD8^+^ depletion experiments. TAMs and other myeloid cells infiltrating tumors suppress antitumor activity in T cells through the secretion of regulatory cytokines like TGFβ, IL‐10, and Arg1 or by overexpressing inhibitory ligands such as PDL1 [46, 47, 48]. We observed substantial increases in CD8^+^ T cell populations within primary and metastatic tumors in *Cxcr2* ^−/−^ or *Cxcr2* ^CD11b‐KO^ mice. Moreover, administering conditioned media from *Cxcr2* ^CD11b‐KO^ TAMs intra‐tumorally significantly reduced tumor growth while augmenting CD8^+^ T cell populations in both primary and metastatic tissues. This supports the hypothesis that *Cxcr2* knock‐out boosts the cytotoxic potential of CD8^+^ T cells by reducing the prevalence and activity of M2 TAMs.

The previous study by Kime et al. demonstrated that the IL‐8/CXCR2 signaling induces M2‐like switching in TAMs through S100A8/A9 level enhancements [43]. Our RNA sequencing analysis of DEGs between BMDMs from WT or *Cxcr2* ^−/−^, *Cxcr2* ^fl/fl^ or *Cxcr2* ^CD11b‐KO^ TAMs, along with co‐culture conditions utilizing CAFs, revealed significant enrichment of DEGs in the PI3K‐AKT‐RhoA signaling pathway. Activation occurs when IL‐8 binds to its receptor, leading to the release of β and γ subunits from G‐protein, which activate downstream signals, including PI3Kγ [49, 50]. The PI3K‐AKT pathway is recognized as a critical regulator influencing macrophage phenotypic transitions [51]. Previous reports indicate that PI3K activation is instrumental for M2 activation in response to agents like surfactant protein A or IL‐4 [52, 53]. Additionally, Akt inhibition disrupts M2 gene expression upregulation [54, 55]. Consistent with these findings, our data suggest that artificial stimulation of the PI3K‐AKT‐RhoA pathway restores M2 polarization in TAMs isolated from *Cxcr2* ^−/−^ or *Cxcr2* ^CD11b‐KO^ mice, whereas blockage of this pathway impairs M2 polarization in RAW264.7 macrophages co‐cultured with CAFs. Extending this, we identified MRTF‐A as a key downstream effector of RhoA in these events. RhoA activation promotes actin polymerization, which releases MRTF‐A from cytoplasmic G‐actin sequestration, enabling its nuclear translocation and subsequent SRF‐dependent transcription [56, 57, 58]. We observed increased nuclear translocation of MRTF‐A in rIL‐8‐treated or CAF‐co‐cultured macrophages, with increased SRF binding. Nuclear MRTF‐A is known to co‐activate SRF to drive target genes, including immunomodulatory genes such as α‐SMA and CTGF [59]. Here, our ChIP‐seq revealed enhanced SRF occupancy at the promoters of M2 genes Arg1 and Mrc1. The MRTF‐A inhibitor CCG‐1423 reversed IL‐8‐driven M2‐like polarization, establishing the RhoA–MRTF‐A–SRF axis as a critical transcriptional driver of immunosuppressive TAM reprogramming downstream of CAF‐derived IL‐8.

For translational relevance, we developed a 3D co‐culture system using PDOs, matched DESMIN^+^ CAFs, and autologous PBMCs, with the addition of PBMC‐derived CD8^+^ T cells. DESMIN^+^ CAFs induced robust M2‐like polarization and CD8^+^ T cell exhaustion, accompanied by MRTF‐A nuclear translocation in human macrophages. CXCR2 blockade with SB225002 attenuated M2 polarization and significantly enhanced autologous CD8^+^ T cell killing of PDOs, mirroring the mechanistic and functional findings in murine models.

Several limitations should be acknowledged. First, although our in vitro models using DESMIN^+^ CAFs, 3LL tumor cells, and RAW264.7 macrophages demonstrated that DESMIN^+^ CAFs are the predominant source of IL‐8, they do not fully exclude potential contributions from other stromal or immune cells in spontaneous human tumors. Second, while the MRTF‐A/SRF axis was identified as a key downstream effector of RhoA in TAMs, its precise contribution relative to other RhoA effectors remains to be fully dissected. Third, although PDO co‐cultures provided valuable human‐relevant validation, larger‐scale studies using primary human TAMs and autologous tumor tissues will be necessary to confirm the therapeutic potential of CXCR2 blockade across diverse patient cohorts.

In conclusion, this study provides compelling evidence that DESMIN^+^ CAFs play a fundamental role in the formation of immunosuppressive TME of LC by promoting M2 polarization of TAMs and the consequent exhaustion of T cells. These effects were achieved, at least in part, through the secretion of IL‐8 and the activation of IL‐8/CXCR2 and the downstream PI3K/AKT/RhoA/MRTF‐A/SRF signaling pathways. Blocking the IL‐8/CXCR2 interaction holds great potential as a strategy to overcome treatment resistance in LC.

## Author Contributions

Xuyu Gu contributed to the conceptualization of the study, the design of the experiments, and the interpretation of the data. Qiyu Fang was involved in the acquisition of data, performing experiments, and drafting the manuscript. Jia Yu participated in the analysis and validation of the results. Ting Yu, Huashan Shi, and Kaiqi Jin provided critical resources and technical support. Lei Jiang supervised the project and contributed to the methodology. Xuyu Gu and Wentian Zhang was responsible for the project administration and funding acquisition. The study was supervised by Kaiqi Jin, Lei Jiang, and Wentian Zhang. All authors reviewed and edited the manuscript, and approved the final version for publication.

## Funding

This study was supported by the Young Scientists Fund of the National Natural Science Foundation of China (82503803 and 82103309).

## Ethics Statement

This study and included experimental procedures were approved by the institutional animal care and use committee of Shanghai Pulmonary Hospital, School of Medicine, Tongji University (Approval NO. K24‐080Y). All animal housing and experiments were conducted in strict accordance with the institutional guidelines for the care and use of laboratory animals.

## Consent

The authors have nothing to report.

## Conflicts of Interest

The authors declare no conflicts of interest.

## Supporting information




**Supporting File**: advs75981‐sup‐0001‐SuppMat.docx.

## Data Availability

Research data supporting this publication are available upon requests.
