# Supplementary material for: Stromal‐Derived IL‐8 Promotes M2 Macrophage Polarization via the RhoA/MRTF‐A/SRF Transcriptional Axis to Impair CD8+ T Cell Cytotoxicity in Lung Cancer
Source: Adv Sci (Weinh). 2026 Jul 16;13(49):e08001. doi: 10.1002/advs.202508001 (PMC13373892; doi:10.1002/advs.202508001)
Supplement: Supplementary file 1 [file ADVS-13-e08001-s001.docx]

**Fig S1** Increased infiltration of DESMIN^+^ cells in stroma correlates with aggressiveness of human LC. A, TMA analysis of DESMIN expression levels in primary tumors and adjacent normal tissues of LC patients; B, Immunofluorescence co-localization analysis of DESMIN and SMA expression in primary tumors and adjacent normal tissues of LC patients; C, Correlation analysis between DESMIN expression levels and pan-myeloid cell markers (CD33, CD14, and CD68) using the TCGA database; D–E, TMA analysis of the correlation between DESMIN staining intensity and the expression of CD14, CD33, and CD68 in LC tissues; F-H, correlations between DESMIN expression and grade (F), lymph node metastases (G) and tumor stage (H) of patients; I, correlations between DESMIN expression and tumor recurrence, lymph node metastasis, tumor differentiation, and microsatellite stability (MSS) of patients; J-L, correlations between the DESMIN staining intensity and the overall survival (J), disease-specific free survival (K), and recurrence-free survival (L) of LC patients; M-N, comparative analysis of Il8 mRNA expression (M) and protein secretion (N) among 3LL cells, RAW264.7 macrophages, and DESMIN^+^/DESMIN^-^ CAFs determined using RT-qPCR and ELISA; O, Expression of active CAF markers FAP and alpha-SMA in isolated DESMIN^+^ CAFs determined using RT-qPCR. Each dot indicates data from one independent experiment. **p* < 0.05, ***p* < 0.001, *****p* < 0.0001.

**Fig S2** CAFs augment tumor progression and metastasis in mice through the IL-8/CXCR2 axis. 3LL cells or the mixture of CAFs and 3LL cells (1:3) were injected into B6 mice. A, serum levels of IL-8, CCL2, CCL5, and CXCL12 in mice determined using ELISA; B, mRNA expression of *Il8* and *Cxcr2* in the formed tumor tissues determined using RT-qPCR; C, protein levels of IL-8 and *Cxcr2* in the tumor tissues analyzed using WB analysis; D-E, CXCR2^+^CD206^+^Ly6C^+^ cells in tumor tissues (D) and bone marrow (E) of mice analyzed using flow cytometry; F-G, TCGA-LUAD data showing the positive correlations between IL-8 and CXCR2 and CAF markers FN1 and VIM; H, schematic presentation of the MTO tumor model and SB225002 or Navarixin treatment; I, the body weight of mice in each group after the traetment; J, number of metastatic nodules in the liver and lymph nodes; K-L, M2-like TAMs (CD206^+^Ly6C^+^) in primary and metastatic tumors determined using flow cytometry. Each dot indicates data from one independent experiment. **p* < 0.05, ***p* < 0.001, ****p* < 0.001, *****p* < 0.0001

**Fig S3** *Cxcr2* deletion reduces tumor metastasis and immune evasion in mice. A, number of tumor nodules in the mouse liver tissue sections; B-C, populations of CD45^+^CD14^+^ (B) and CD45^+^CD33^+^ (C) in the liver metastatic tumor sections determined using flow cytometry; D-E, populations of CD206^+^Ly6C^+^ (D) or CD86^+^Ly6C^+^ (E) cells in the liver metastatic tumor sections determined using flow cytometry; F-I, populations of PD-1^+^ CD8^+^ T (F), TIM3^+^CD8^+^ T (G), GZMB^+^CD8^+^ T (H), and TNFA^+^ CD8^+^ T (JI) cells in the orthotopic tumor tissues determined using flow cytometry. J-K, positive staining of CD31 (J), and VEGFA (K) in the liver metastatic tumor sections determined using IHC. Each dot indicates data from one independent experiment. ***p* < 0.01, ****p* < 0.001, *****p* < 0.0001.

**Fig S4** The IL-8 monoclonal antibody diminishes the promoting effect of DESMIN-CAFs on immunosuppressive polarization of RAW264.7 macrophages. A, schematic illustration of the co-culture system of mouse RAW264.7 macrophages and DESMIN^+^ CAFs, and the addition of IL-8 monoclonal antibody; B, proportion of CD206^+^Ly6C^+^ macrophages after CAF stimulation or anti-IL-8 treatment determined using flow cytometry; C, mRNA expression of CD206, CD36, and Arg1 in macrophages determined using qPCR analysis. D, schematic illustration of the co-culture system of mouse RAW264.7 macrophages and DESMIN^+^ CAFs, and the addition of CXCR2 neutralizing antibody; E-F, mRNA (E) and protein (F) levels of CD206, CD36, CD86, and iNOS in the RAW264.7 cells after co-culture determined using RT-qPCR and WB analysis; G, fluorescence intensity of Arg1 on the surface of RAW264.7 cells determined using immunofluorescence staining; H, concentrations of TNFα, IL-6, TGFβ, and CSF1 in the co-culture system determined using ELISA kits; I, migration of RAW264.7 macrophages determined using Transwell assays; J, schematic illustration of the culture system of mouse RAW264.7 macrophages and the addition of rIL-8 or anti-CXCR2; K, population of CD206^+^Ly6C^+^ cells determined using flow cytometry; L, concentrations of CSF1, TGFβ, and VEGFA in the culture system determined using ELISA kits. M, PMA-treated THP-1 cells co-cultured with IL-8, followed by the addition of CXCR1 high-affinity inhibitor Reparixin or the CXCR2 inhibitor SB225002; N, population of CD206^+^Arg^+^ macrophages determined using flow cytometry; O, concentrations of TGFβ and Arg1 in the culture system determined using ELISA kits. Each dot indicates one independent experiment. **p* < 0.05, ***p* < 0.001, ****p* < 0.001, *****p* < 0.0001.

**Fig S5** Knock-out of *Cxcr2* in mouse myeloid cells enhances immune activity in the liver metastatic tumors. A, population of CD206^+^Ly6C^+^ cells in the liver metastatic tumor tissues determined using flow cytometry; B-E, positive staining of PD-1 (B), PD-L1 (C), CTLA4 (D), and GAL-9 (E) in the liver metastatic tissues determined using IHC; F-G, populations of GZMB^+^CD8^+^ T cells or VISTA^+^CD8^+^ T cells in the liver metastatic tumor tissues determined using immunofluorescence staining. H, schematic illustration of the BMDM collection and treatment; BMDMs from *Cxcr2*^fl/fl^ mice were extracted, stimulated with m-CSF and IL-4 to obtain a TAM phenotype, followed by treatments of rIL-8, or SB225002; I-J, populations of CD206^+^Ly6C^+^ (I) or CD86^+^Ly6C^+^ (J) cells determined using flow cytometry; K, concentrations of IL-4, TGFβ, and VEGFA in the culture system determined using ELISA kits. Each dot indicates one independent experiment. ***p* < 0.01, ****p* < 0.001, *****p* < 0.0001.

**Fig S6** Loss of *Cxcr2* improves immune activity of CD8^+^ T cells in metastatic tumor tissues. A-B, population of CD8^+^ T cells in the metastatic tumors of each group of mice determined using immunofluorescence staining; C-D, populations of TCF7^+^, CTLA-4^+^, CD69^+^, IFNγ^+^, and IL-1^+^ CD8^+^T cells in the metastatic tumors of mice determined using flow cytometry; E-F, mRNA and protein levels of CD8^+^ T cell dysfunction markers (TIM-3, LAG-3, CD38, 2B4, and TBX21) and stem-like CD8 T cell signatures (TCF7, CD122, CD127, CD45RA, and CCR7) in the metastatic tumors determined using RT-qPCR and WB analysis. G, schematic illustration of the subcutaneous tumor model; mouse 3LL cells were injected into *Cxcr2*^fl/fl^ or *Cxcr2*^CD11b-KO^ mice subcutaneously; H-J, volume and weight of the subcutaneous tumors in mice; K, schematic illustration of another subcutaneous tumor model; mouse 3LL cells were mixed with TAMs derived from *Cxcr2*^fl/fl^ or *Cxcr2*^CD11b-KO^ mice and then injected into B6 recipient mice subcutaneously; L-N, volume and weight of the subcutaneous tumors in mice. Each dot indicates one independent experiment. **p* < 0.05, ***p* < 0.001, ****p* < 0.001, *****p* < 0.0001.

**Fig S7** Intratumoral administration of CM of TAMs affects tumor growth in mice. A, schematic illustration of the animal treatment; TAMs derived from either *Cxcr2*^fl/fl^ or *Cxcr2*^CD11b-ko^ mice were collected and cultured, and the CM was injected into B6 mice bearing subcutaneous tumors formed by 3LL cells; B-D, volume and weight of the subcutaneous tumors in mice; E, schematic illustration of the animal treatment; TAMs derived from either *Cxcr2*^fl/fl^ or *Cxcr2*^CD11b-ko^ mice were collected and cultured, and the CM was injected into B6 mice bearing subcutaneous tumors formed by KLN205 cells; F-H, volume and weight of the subcutaneous tumors in mice. I, schematic illustration of the modeling process; control RAW264.7 cells or RAW264.7 cells overexpressing Cxcr2 were stimulated with IFNγ and LPS to induce an M1 phenotype. The CM was collected, mixed with 3LL cells, and implanted into B6 mice; J-L, volume and weight of the subcutaneous tumors in mice; M, the body weight of mice in each group after the treatment; N, positive staining of KI67 and PCNA in the tumor tissues determined using IHC. Each dot indicates one independent experiment. ****p* < 0.001, *****p* < 0.001.

**Fig S8** *Cxcr2* deletion in TAMs reduces tumor growth and metastasis in immunocompromised mice. A, schematic illustration of the modeling process; TAMs were extracted from *Cxcr2*^fl/fl^ and *Cxcr2*^CD11b-KO^ mice using magnetic cell sorting, which were mixed with 3LL cells and injected into T cell-deficient BALB/c nude mice; B-D, volume and weight of the subcutaneous tumors in mice; E, positive staining of KI67 and PCNA in the tumor tissues determined using IHC. E, schematic presentation of the experimental setting, luciferase-labeled KLN205 cells were administered along with TAM-derived CM to BALB/nude mice via splenic injection, and TAM-derived CM was additionally intraperitoneally; F, representative bioluminescence images and statistical data of bioluminescence signal; G, the body weight of mice in each group after the treatment; H, liver lobes examined by HE staining and number of lobes with tumor; I, liver-to-body weight ratio. Each dot indicates data from one independent experiment. ****p* < 0.001, *****p* < 0.0001.

**Fig S9 MRTF-A/SRF signaling axis is responsible for IL-8-triggered M2 polarization of TAMs.** A-B, Nuclear translocation of MRTF-A in RAW264.7 macrophages or BMDMs treated with rIL-8 (10 ng/mL) or co-cultured with DESMIN^+^ CAFs determined using immunofluorescence staining (A) and Western blot analysis of cytoplasmic and nuclear fractions (B);C-D, Global enrichment of SRF binding sites (C) and heatmap of SRF-occupied genomic regions (D) in macrophages treated with or without rIL-8 determined using ChIP-seq analysis; E, Representative ChIP-seq tracks (IGV snapshots) showing the enrichment of SRF occupancy at the promoter regions of Arg1 and Mrc1 in macrophages following rIL-8 stimulation; F-H, Populations of CD206^+^Ly6C^+^M2-like macrophages (G) and mRNA expression levels of M2 markers (H) in macrophages treated with rIL-8 in the presence or absence of the MRTF-A specific inhibitor CCG-1423 (0.3 μM) determined using flow cytometry and RT-qPCR. Each dot indicates data from one independent experiment. **** *p* < 0.0001.

**Fig S10 Reconstitution of human lung cancer immune microenvironment using a PDO tri-culture system.** A, Schematic illustration of the experimental design for the human lung cancer PDO tri-culture system. PDOs were co-cultured with patient-matched DESMIN^+^ CAFs and peripheral blood-derived monocytes in 3D Matrigel, followed by the addition of autologous CD8^+^ T cells and treatment with or without SB225002 (1 μM); B, Proportions of CD206^+^Ly6C^+^ M2-like macrophages in the PDO tri-culture system under different conditions determined using flow cytometry; C-D, Proportions of GZMB^+^ (Cytotocixity) and Tim3^+^PD-1^+^ (exhausted) CD8^+^ T cells in the tri-culture system determined using flow cytometry. E, Nuclear translocation of MRTF-A in human primary macrophages within the PDO tri-culture system in the presence or absence of DESMIN^+^ CAFs and SB225002 determined using immunofluorescence staining. Each dot indicates one independent experiment. * p < 0.05, ** p < 0.01, *** p < 0.001, **** p < 0.0001.

**Key resource table**

| **Item/Reagent** | **Concentration/Quantity** | **Source/Supplier** | **Catalog/** |
| --- | --- | --- | --- |
| **Cell culture and treatment material** | | | |
| Collagenase type XI | 0.5 mg/mL | Sigma Aldrich, St. Louis, MO | C9407 |
| Dispase | 0.2 mg/mL | Sigma Aldrich | 17105041 |
| Advanced DMEM/F12 | - | Invitrogen, Waltham, MA | - |
| Matrigel | - | BD Bioscience, Basel, Halbkanton | 12634010 |
| Penicillin/streptomycin | 1% | Life Technologies | - |
| GlutaMAX | 1% | Life Technologies | 15140122 |
| HEPES | 10 mmol/L | Life Technologies | BE-17-605E/U1 |
| B27 | 2% vol/vol | Life Technologies | be-17-737E |
| N2 | 1% vol/vol | Life Technologies | 17504-001 |
| N-acetylcysteine | 1.25 μmol/L | Sigma-Aldrich | 17502001 |
| Gastrin | 10 nmol/L | Sigma Aldrich | A7250 |
| EGF | 50 ng/mL | PeproTech, London | G9145 |
| Freeze medium | - | - | Y0503 |
| Collagenase type XI | 0.5 mg/mL | Sigma Aldrich | - |
| Dispase | 0.2 mg/mL | Sigma Aldrich | - |
| Fetal bovine serum | 1% | Life Technologies | - |
| Advanced DMEM/F12 | - | Invitrogen |  |
| Matrigel | - | BD Bioscience | - |
| Penicillin/streptomycin | 1% | Life Technologies | - |
| GlutaMAX | 1% | Life Technologies | - |
| HEPES | 10 mmol/L | Westburg BV |  |
| SB225002 |  |  |  |
| Navarixin |  |  |  |
| **Antibodies and commercial kit for WB, IHC and IF** | | | |
| αCD8 antibody | 200 μg | BioXcell |  |
| IgG | 200 μg | BioXcell |  |
| Tumor Dissociation Kit | 1 kit | Miltenyi Biotec |  |
| Red Blood Cell Lysis Solution | 1 bottle | Miltenyi Biotec | 130-094-183 |
| CD3ε MicroBead Kit | 1 kit | Miltenyi Biotec | 130-094-973 |
| Anti-CD3/CD28 antibodies | - | Thermo Fisher | 11452D |
| Protein transport inhibitor | - | BD Biosciences | 554724 |
| CD3 Antibody | 1:70 | BD Biosciences | 555275 |
| CD8 Antibody | 1:70 | Biolegend | 100706 |
| CD69 Antibody | 1:70 | Biolegend | 104512 |
| IFNg Antibody | 1:70 | BD Biosciences | 563376 |
| TCF7 Antibody | 1:70 | BD Biosciences | 566692 |
| PDCD1 Antibody | 1:70 | Biolegend | 135224 |
| CTLA4 Antibody | 1:70 | Biolegend | 106314 |
| Biotinylated or Fluorophore-conjugated Secondary Antibody | 1:200 | BA-5000, BA-1000, BA-2000, BA-4000 (for biotinylated) | Vector Laboratories |
| Avidin-Biotin Complex | - | Vectastain Elite | Vector Laboratories |
| Alexa Flour 488 or 594 Conjugated Secondary Antibodies | 1:200 | A21207, A21203, A21202, A21206 | Thermo Fisher |
| Opal 3-Plex Manual Kit | - | - | Akoya Biosciences |
| Secondary Antibodies (ImmPRESS Reagent, EnVision+ System-HRP Labelled Polymer) | Undiluted | MP-7401, MP-7404, K4001 | Vector Laboratories, Dako |
| DESMIN | 1:200 | ab32362 | Abcam |
| CD31 | 1:100 | 7769 | Cell Signaling |
| CD8 | 1:2000 | ab209775 | Abcam |
| CD4 | 1:1000 | ab183685 | Abcam |
| CD11c | 1:1000 | 97585 | Cell Signaling |
| C-Cas3 | 1:100 | 9664 | Cell Signaling |
| CXCR2 | 1:500 | ab52625 | Abcam |
| IL-8 | 1:1000 | ab133357 | Abcam |
| EPCAM | 1:100 | ab71916 | Abcam |
| Ly6C | 1:100 | 128002 | Biolegend |
| Ly6G | 1:1000 | 87048 | Cell Signaling |
| F4/80 | 1:2500 | 70076 | Cell Signaling |
| FOXP3 | 1:1000 | 12653 | Cell Signaling |
| TCF7 | 1:1500 | MA514965 | Thermo Fisher |
| CD36 | 1:1500 | ab202909 | Abcam |
| CTLA4 | 1:1000 | ab237712 | Abcam |
| PCNA | 1:500 | ab92552 | Abcam |
| KI67 | 1:500 | ab16667 | Abcam |
| CXCR2 | 1:1000 |  |  |
| IL8 | 1:1000 |  |  |
| **Antibodies for flow cytometry** | | | |
| CD45 | 1:70 | 552848 | BD Biosciences |
| CD11b | 1:70 | 553310 | BD Biosciences |
| CXCR4 | 1:70 | 146511 | BioLegend |
| EPCAM | 1:70 | 48-5791-82 | Invitrogen |
| CD45 | 1:70 | 557659 | BD Biosciences |
| CD45 | 1:70 | 103106 | BioLegend |
| CD3 | 1:70 | 555275 | BD Biosciences |
| CD3 | 1:70 | 100236 | BioLegend |
| CD4 | 1:70 | 100406 | BioLegend |
| CD8 | 1:70 | 553035 | BD Biosciences |
| CD8 | 1:70 | 100706 | BioLegend |
| CD69 | 1:70 | 104512 | BioLegend |
| CD11c | 1:70 | 117318 | BioLegend |
| CD11b | 1:70 | 553310 | BD Biosciences |
| F4/80 | 1:70 | 565410 | BD Biosciences |
| Ly6C | 1:70 | 560595 | BD Biosciences |
| Ly6G | 1:70 | 562737 | BD Biosciences |
| PDCD1 | 1:70 | 135224 | BioLegend |
| CTLA4 | 1:70 | 106314 | BioLegend |
| CCR2 | 1:70 | 150610 | BioLegend |
